# NSG-Pro mouse model for uncovering resistance mechanisms and unique vulnerabilities in human luminal breast cancers

**DOI:** 10.1126/sciadv.abc8145

**Published:** 2021-09-15

**Authors:** Yunguang Sun, Ning Yang, Fransiscus E. Utama, Sameer S. Udhane, Junling Zhang, Amy R. Peck, Alicia Yanac, Katherine Duffey, John F. Langenheim, Vindhya Udhane, Guanjun Xia, Jess F. Peterson, Julie M. Jorns, Marja T. Nevalainen, Romain Rouet, Peter Schofield, Daniel Christ, Christopher J. Ormandy, Anne L. Rosenberg, Inna Chervoneva, Shirng-Wern Tsaih, Michael J. Flister, Serge Y. Fuchs, Kay-Uwe Wagner, Hallgeir Rui

**Affiliations:** 1 Department of Pathology, Medical College of Wisconsin, Milwaukee, WI 53226, USA.; 2 Department of Pharmacology and Toxicology, Medical College of Wisconsin, Milwaukee, WI 53226, USA.; 3 Immunology Division, University of New South Wales, Darlinghurst, NSW 2010, Australia.; 4 Garvan Institute of Medical Research and St. Vincent’s Clinical School, University of New South Wales, Darlinghurst, NSW 2010, Australia; 5 Department of Surgery, Thomas Jefferson University, Philadelphia, PA 19107, USA.; 6 Department of Pharmacology, Division of Biostatistics, Thomas Jefferson University, Philadelphia, PA 19107, USA.; 7 Department of Physiology, Medical College of Wisconsin, Milwaukee, WI 53226, USA.; 8 Department of Biomedical Sciences, University of Pennsylvania School of Veterinary Medicine, Philadelphia, PA 19104, USA.; 9 Karmanos Cancer Institute, Wayne State University, Detroit, MI 48201, USA.

## Abstract

Most breast cancer deaths are caused by estrogen receptor-α–positive (ER^+^) disease. Preclinical progress is hampered by a shortage of therapy-naïve ER^+^ tumor models that recapitulate metastatic progression and clinically relevant therapy resistance. Human prolactin (hPRL) is a risk factor for primary and metastatic ER^+^ breast cancer. Because mouse prolactin fails to activate hPRL receptors, we developed a prolactin-humanized Nod-SCID-IL2Rγ (NSG) mouse (NSG-Pro) with physiological hPRL levels. Here, we show that NSG-Pro mice facilitate establishment of therapy-naïve, estrogen-dependent PDX tumors that progress to lethal metastatic disease. Preclinical trials provide first-in-mouse efficacy of pharmacological hPRL suppression on residual ER^+^ human breast cancer metastases and document divergent biology and drug responsiveness of tumors grown in NSG-Pro versus NSG mice. Oncogenomic analyses of PDX lines in NSG-Pro mice revealed clinically relevant therapy-resistance mechanisms and unexpected, potently actionable vulnerabilities such as DNA-repair aberrations. The NSG-Pro mouse unlocks previously inaccessible precision medicine approaches for ER^+^ breast cancers.

## Teaser

Prolactin-humanized mice promote growth and metastatic progression of estrogen-dependent patient-derived breast cancer.

## INTRODUCTION

The highest burden of breast cancer deaths is due to luminal tumors (70 to 80% of all newly diagnosed cases) that later recur as metastatic and therapy-resistant disease [~20% of the initial estrogen receptor-α–positive (ER^+^) cases] (*1*, *2*). Thus, the paucity of metastasizing, estrogen-dependent models of human ER^+^ breast cancer is a pressing issue. While the mouse mammary intraductal model of breast cancer cell instillation (*3*) provides a more supportive growth environment for human ER^+^ tumor cells than does orthotopic mammary implantation (*4*), invasion through the basement membrane is limited, and macrometastatic progression models have not been accomplished, with only occasional distant metastatic cells observed (*5*). The mechanisms leading to therapy resistance in ER^+^ breast cancer are numerous and differ between individual tumors because of patient-specific somatic driver mutations, copy number variation, and epigenetic factors (*6*–*8*). One strategy to personalize anticancer therapy involves identifying drug responsiveness and mechanisms of therapy resistance using patient-derived xenograft (PDX) models (*9*).

An advantage of PDX models is that unique characteristics of a patient’s tumor tissue can be biologically replicated across multiple animals, enabling drug testing and mechanistic studies in vivo. However, a major weakness of current breast cancer PDX models is the limited diversity and lack of therapy-naïve, metastasizing ER^+^ models (*10*, *11*). The vast majority of estrogen-dependent breast cancers do not grow or metastasize in conventional PDX host strains (*12*, *13*), with the largest study reporting a 2.5% engraftment rate of therapy-naïve luminal ER^+^ breast tumor xenografts (*13*). ER^+^ PDX models from treated patients are selected for estrogen-independent tumors, including drug-resistant tumors with constitutively active ER mutations (*14*) that are not detected in therapy-naïve primary breast cancers (*15*).

The pituitary hormone prolactin (PRL) is essential for proliferative expansion of normal differentiated breast epithelial cells (*16*), promotes malignant ER^+^ mammary tumors in rodents (*17*–*20*), and is a risk factor for ER^+^ breast cancer in women (*17*, *18*, *21*–*23*). Several lines of mechanistic evidence support a synergistic interaction between PRL and estrogen signaling in human breast cancer (*24*, *25*). Emerging evidence also support prometastatic roles of PRL for ER^+^ breast cancer (*26*, *27*). We previously demonstrated that mouse PRL (mPRL) does not activate human PRL receptor (hPRLR) (*28*) and, in fact, acts as an hPRLR antagonist (*29*). On the basis of these data, we hypothesized that the lack of hPRL in mouse hosts deprives xenografted human ER^+^ breast cancers of an essential endocrine factor that governs tumor engraftment, growth, metastasis, and drug responsiveness (*29*, *30*). We reasoned that a PRL-humanized PDX host strain should support engraftment and metastasis of luminal ER^+^ breast cancer while maintaining high engraftment rates of ER-negative breast cancer subtypes that may be less dependent on PRL.

Here, we show that the PRL-humanized Nod-*scid*-IL2Rγ (NSG) (NSG-Pro) mouse, a unique host with physiological levels of circulating hPRL in the absence of mPRL, supports the engraftment, growth, metastasis, and therapeutic responses of all common breast cancer subtypes. This NSG-Pro model enables a 15- to 20-fold improvement in engraftment rates of therapy-naïve ER^+^ breast tumors and faithfully recapitulates the transition of these tumors to spontaneously metastatic and therapy-resistant disease. Comprehensive biological, transcriptomic, and genomic characterization also revealed that physiological levels of hPRL were critical for ER^+^ PDX to fully manifest patient-specific therapeutic targets, including the development of aberrant ER signaling and nongenomic ERBB2 up-regulation following tamoxifen treatment. Additional studies of ER^+^ PDX in the NSG-Pro model aided the identification of otherwise underappreciated genetic alterations that became selectively amplified in tamoxifen-resistant tumors and predisposed these resistant derivatives to remarkable responses to poly(adenosine diphosphate–ribose) polymerase (PARP) and DNA ligase 3 (LIG3) inhibitors.

## RESULTS

### Human but not murine PRL stimulates proliferation of human breast cancer cells

**Fig. 1.**
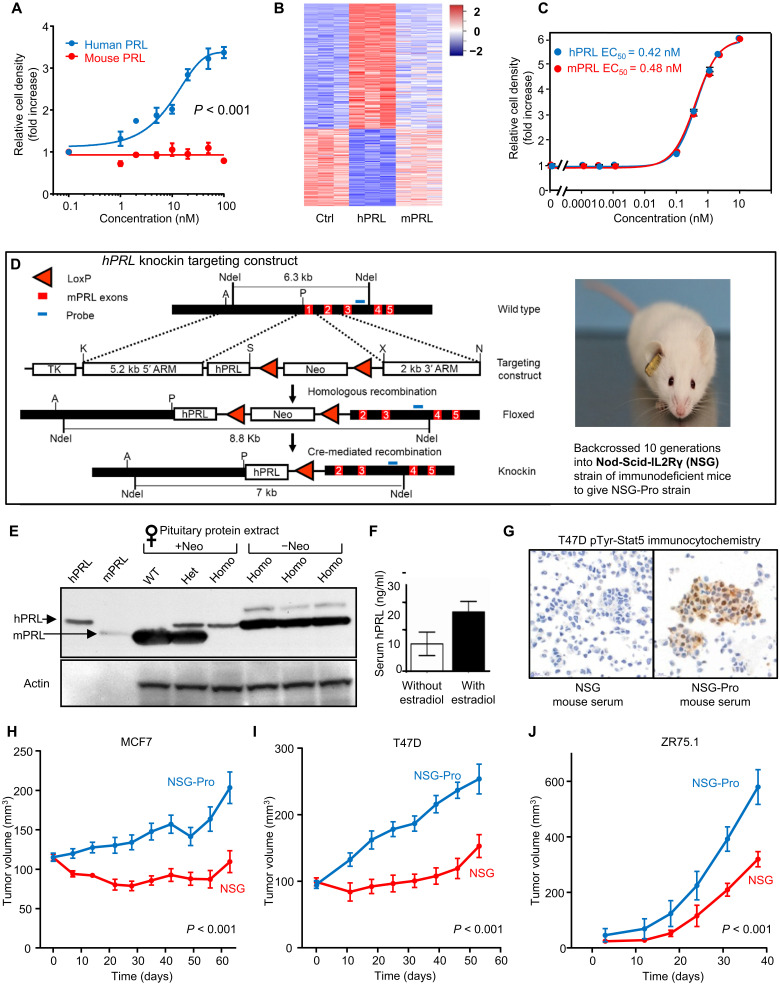
Generation and initial validation of NSG-Pro mouse model. (**A**) Proliferation of human T47D breast cancer cells in 3D spheroid culture treated with increasing concentrations of hPRL or mPRL. (**B**) Heatmap of up- and down-regulated genes in T47D cells in 2D culture in response to hPRL versus mPRL compared to untreated controls (Ctrl). (**C**) Proliferation of murine 32D myeloid cells stably transfected with hPRLR treated with increasing concentrations of hPRL or mPRL. EC_50_, median effective concentration. (**D**) Schematic representation of *hPRL* knockin targeting construct and the final gene structure of the knockin *hPRL* gene locus. TK, *thymidine kinase*. Photo of NSG-Pro mouse (photo credit: Yunguang Sun, Medical College of Wisconsin, Milwaukee, WI). (**E**) Western blot of PRL from pituitary extracts of wild-type (WT) mice and hPRL-knockin mice (Het, heterozygous; Homo, homozygous) before and after removal of the floxed neomycin (neo) cassette, using a polyclonal PRL antibody that recognizes both hPRL and mPRL. (**F**) Serum hPRL levels in female NSG-Pro mice with or without slow-release 17β-estradiol pellets. (**G**) Phosphotyrosine-Stat5 immunocytochemistry of T47D cells treated with or without 50% serum from NSG or NSG-Pro mice. (**H**) Growth curves of MCF7 breast cancer xenograft tumors in NSG-Pro versus NSG mice. (**I**) Growth curves of T47D breast cancer xenograft tumors in NSG-Pro versus NSG mice. (**J**) Growth curves of ZR75.1 breast cancer xenograft tumors in NSG-Pro versus NSG mice.

Previous reports suggested that mPRL is a poor ligand for hPRLR (*28*, *29*). We indeed found that exposure to hPRL, but not mPRL, drove the proliferative expansion of human T47D breast cancer three-dimensional (3D) spheroids in a dose-dependent manner (Fig. 1A). Parallel RNA sequencing (RNA-seq) analyses of 2D cultures of T47D cells revealed extensive differential modulation of gene expression by hPRL [differentially expressed (DE) genes = 1076, false discovery rate (FDR) < 0.05] but not mPRL (DE genes = 5, FDR < 0.05; Fig. 1B and table S1). Moreover, only hPRL modulated expression of gene networks that are downstream of the hPRLR (*z* score = 2.9, *P* = 2 × 10^−12^; fig. S1A) as well as downstream targets of ERBB2 (*z* score = 3.3, *P* = 5.6 × 10^−31^; fig. S1B) and of ER (*z* score = 3.9, *P* = 4 × 10^−31^; fig. S1C), which are key pathways broadly implicated in malignant breast epithelial growth and differentiation (*31*, *32*). In contrast to the inability of mPRL to activate hPRLR-dependent signaling and proliferation in T47D cells (Fig. 1, A and B), hPRL and mPRL induced equipotent proliferative responses in mPRLR^+^ 32D murine cancer cells (Fig. 1C). On the basis of our observations and the recognized cooperative interactions between PRL and ER signaling in breast cancer (*24*, *25*), we hypothesized that inadequacy of the PRL/PRLR signaling axis is a major contributing cause of the poor human-in-mouse engraftment rates of ER^+^ PDX models.

### Development of the NSG-Pro mouse

To develop a PDX host strain with physiological levels of circulating hPRL, we generated prolactin-humanized mice by knock-in of the coding sequence of the full-length human *PRL* into the first exon of the *Prl* gene in 129/Sv mouse embryonic stem cells (Fig. 1D). Following chimera production and validated germline transmission of the targeted *hPRL-Prl* allele, we intercrossed the knockin mice with the MMTV-Cre (line A) strain, which expresses Cre recombinase in developing oocytes (*33*), to remove the floxed neomycin selection cassette and to transcriptionally activate the *hPRL* from the endogenous mouse *Prl* locus. F_2_ offspring were then backcrossed for 10 generations into the NSG background, and we generated immunocompromised NSG mice that are homozygous for the targeted *hPRL-Prl* allele (NSG-Pro). Immunoblot analysis revealed that these animals expressed only the hPRL in the complete absence of the mouse hormone in their pituitary glands (Fig. 1E). Circulating hPRL in female NSG-Pro mice averaged 10 ng/ml and increased twofold in response to estrogen (Fig. 1F), consistent with intact physiological estrogen-driven pituitary *PRL* gene expression (*34*). There was no evidence of hyperprolactinemia (hPRL >30 ng/ml) in the NSG-Pro mouse, and the levels of circulating hPRL closely parallel those in healthy nonpregnant women, demonstrating the physiological relevance of the new NSG-Pro model as an appropriate host for PRLR-expressing human tumors and tissues. We tested the biological activity of circulating hPRL by exposing human T47D breast cancer cells to serum from female NSG-Pro or conventional NSG mice as controls. Only the NSG-Pro serum was able to induce the tyrosine phosphorylation and nuclear localization of the signal transducer and activator of transcription-5 (STAT5), which is an established intermediary of hPRLR signaling in these cells (Fig. 1G). In a biological assay, three of the three cell line–based ER^+^ breast cancer xenografts (MCF7, T47D, and ZR75.1) tested grew significantly better in the novel humanized NSG-Pro strain compared to nonhumanized NSG mice (Fig. 1, H to J). Naïve homozygous NSG-Pro mice appear healthy and display no overt pathology phenotypes compared to NSG mice. There was no reduction in growth of human triple-negative breast cancer (TNBC) PDX tumors with undetectable hPRLR expression in hPRL-humanized compared to nonhumanized recipient animals (fig. S1D and table S2). Collectively, the data demonstrate that the NSG-Pro mouse model provides physiological levels of hPRL and improves the growth of common human ER^+^ breast cancer cell lines as xenografts while retaining the ability of NSG mice to host ER-negative tumors.

### Substantially improved PDX engraftment rates in NSG-Pro mice

**Fig. 2.**
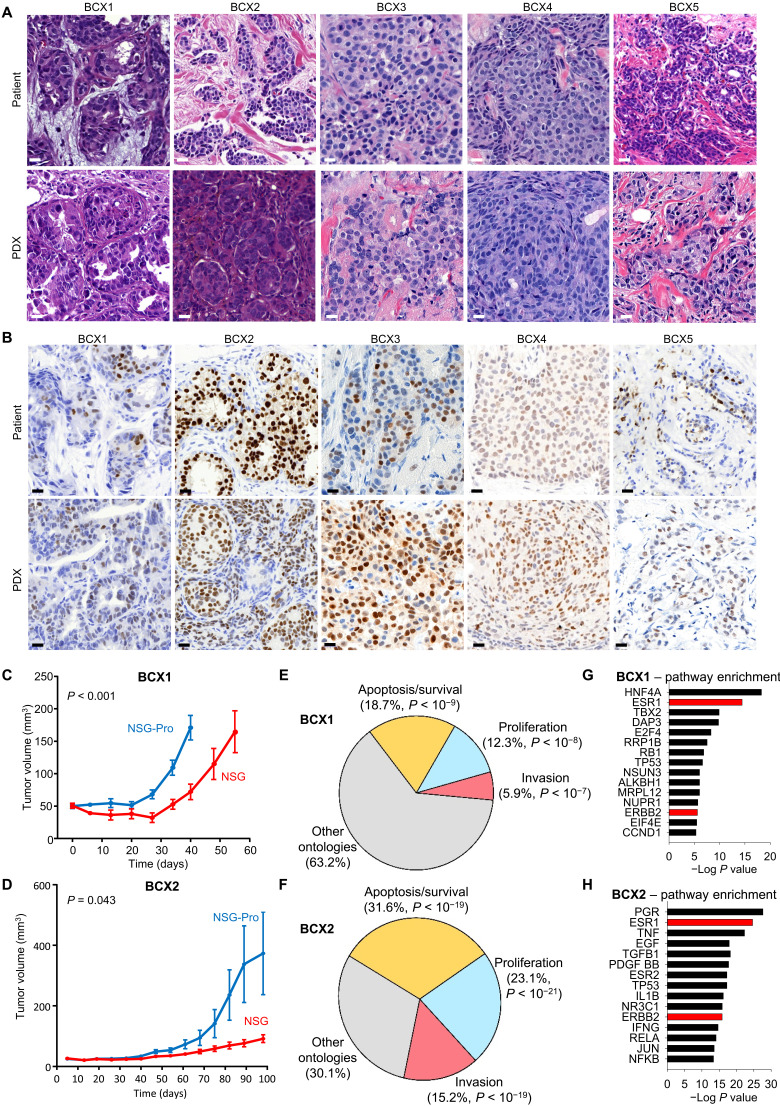
Histology of ER^+^ PDX models of breast cancer. (**A**) Histology of five serially transplantable ER^+^ breast cancer PDX tumors BCX1 to BCX5 as propagated in NSG-Pro mice compared with original patient tumors. Scale bars, 20 μm. (**B**) Immunohistochemistry of ER in BCX1 to BCX5 PDX models grown in NSG-Pro mice and original patient tumors using diaminobenzidine stain (brown) as chromogen. Percent ER positivity of patient tumor versus PDX model were generally consistent: BCX1 (14% versus 20%), BCX2 (64% versus 67%), BCX3 (20% versus 80%), BCX4 (64% versus 69%), and BCX5 (32% versus 18%). Scale bars, 20 μm. (**C**) Growth curves of BCX1 PDX breast tumors in NSG-Pro versus NSG mice. (**D**) Growth curves of BCX2 PDX breast tumors in NSG-Pro versus NSG mice. (**E**) Gene ontologies modulated by hPRL as revealed by human-specific RNA-seq of BCX1 tumors propagated in NSG-Pro mice versus NSG mice. (**F**) Gene ontologies modulated by hPRL as revealed by human-specific RNA-seq of BCX2 tumors propagated in NSG-Pro mice versus NSG mice. (**G**) Gene pathway enrichment in BCX1 tumors propagated in NSG-Pro mice versus NSG mice as revealed by human-specific RNA-seq. (**H**) Gene pathway enrichment in BCX2 tumors propagated in NSG-Pro mice versus NSG mice as revealed by human-specific RNA-seq.

Analysis of surgical specimens available from 65 consecutive patients with invasive therapy-naïve primary ER^+^ breast cancer revealed an engraftment rate of 43% (28 of 65 tumors) in the NSG-Pro mouse (table S2), which is 17 times higher than the 2.5% previously reported in the largest study using nonhumanized recipient mice (*10*). Successful engraftment of a PDX was defined as doubling in size or more than three serial passages of tumors. PDX tumors grown in NSG-Pro mice recapitulated the activation pattern of STAT5 in the primary patient tumor (fig. S1E) and generally recapitulated the histological characteristics, Ki67 levels, and hormone receptor expression status [ER, PR (progesterone receptor), and ERBB2] as shown for representative patient tumors (BCX1 to BCX5; Fig. 2, A and B, and fig. S2). Similar to ER^+^ breast cancer cell line xenografts (Fig. 1, H to J), established ER^+^ luminal PDX tumors BCX1 and BCX2 grew significantly faster in the NSG-Pro mouse model compared to conventional NSG mice (Fig. 2, C and D), further demonstrating that the physiological levels of hPRL in the NSG-Pro model support the engraftment and the continuous growth of ER^+^ PDXs. The NSG-Pro model also supports high engraftment rates of the ERBB2-enriched (40%; two of five tumors) and TNBC (57%; four of seven tumors) subtypes (table S2). While luminal subtypes express higher PRLR levels, PRL signaling may also be relevant for ER-negative ERBB2-enriched and certain TNBC subtypes as supported by their frequent expression of PRLR transcripts according to The Cancer Genome Atlas (TCGA) data (fig. S3A). Thus, the NSG-Pro mouse model enables the development of a diverse breast cancer PDX library and, therefore, faithfully recapitulates the current spectrum of patients with distinct tumor subtypes.

To determine hPRL/PRLR-dependent pathways that may facilitate the enhanced engraftment rates of ER^+^ PDX in the NSG-Pro mouse model, we performed RNA-seq in human ER^+^ tumors propagated in NSG-Pro mice or conventional NSG animals. The study revealed that in the BCX1 and BCX2 tumors, a total of 6416 and 801 human genes, respectively, were DE in the two hosts (tables S3 and S4). Gene network analysis showed significant enrichment of key breast cancer gene ontologies in the NSG-Pro model. These included pathways mediating tumor cell proliferation, survival, and invasion (Fig. 2, E and F) as well as several essential molecular pathways that are known to be critical for the pathophysiology of ER^+^ breast cancers, in particular, the ESR1 and ERBB2 pathways (Fig. 2, G and H). Collectively, these data suggest that physiological levels of hPRL in the NSG-Pro mouse model control key breast cancer–related pathways that are critical for the engraftment, growth, and maintenance of ER^+^ breast cancer PDX.

### The NSG-Pro model facilitates the metastatic progression of ER^+^ PDX

**Fig. 3.**
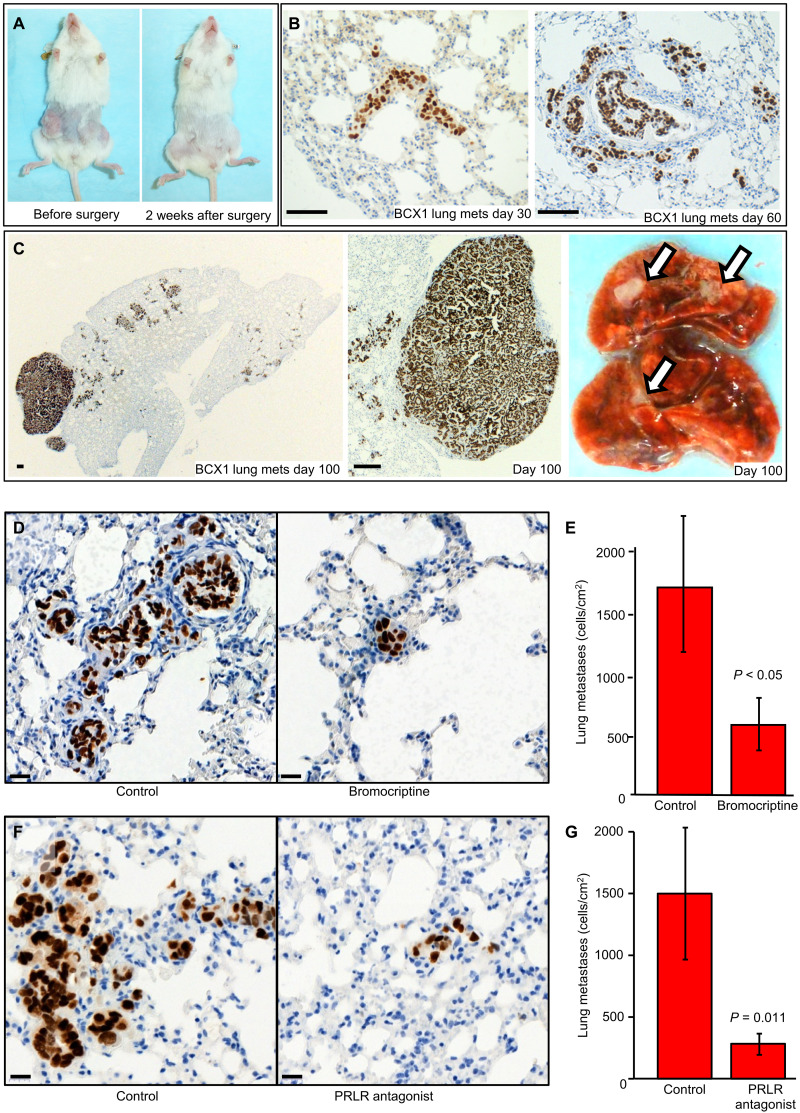
ER^+^ breast cancer PDX models that spontaneously metastasize to distant sites in NSG-Pro mice. (**A**) Orthotopically implanted BCX1 tumors grown in the fourth paired mammary glands of an NSG-Pro mouse for 55 days and the same mouse shown 2 weeks after surgical removal of tumors (photo credit: Ning Yang, Medical College of Wisconsin, Milwaukee, WI). (**B**) Representative lung metastases of BCX1 as visualized by human-specific mitochondrial protein marker (brown) at 30 and 60 days after surgical ablation of primary tumors. Scale bars, 200 μm. (**C**) Representative macrometastatic lung lesion metastases of BCX1 at 100 days after surgical ablation of primary tumors as visualized by human-specific mitochondrial protein marker (brown) or as white lesions (arrows) in whole lung photo. Scale bars, 200 μm. (**D**) Representative images of lung metastases of BCX1 in control or bromocriptine-treated NSG-Pro mice as visualized by human-specific mitochondrial protein marker (brown). Scale bars, 200 μm. (**E**) Quantitative analysis of lung metastases of BCX1 in NSG-Pro mice treated with bromocriptine or vehicle for 14 days as visualized by human-specific mitochondrial protein marker (brown). Scale bars, 200 μm. (**F**) Representative images of lung metastases of BCX1 in NSG-Pro mice treated with antagonist anti-PRLR antibody or vehicle for 10 days as visualized by human-specific mitochondrial protein marker (brown). Scale bars, 200 μm. (**G**) Quantitative analysis of lung metastases of BCX1 in control or anti-PRLR antibody–treated NSG-Pro mice.

Another critical impediment to studying the biology and therapy responses of ER^+^ human breast cancer is the lack of orthotopic models that spontaneously metastasize to vital organs and recapitulate progression to macrometastatic and fatal disease (*12*). To examine whether the NSG-Pro mouse could facilitate the metastatic dissemination of ER^+^ PDX tumors, we orthotopically implanted mice with the ER^+^ PDX lines BCX1, BCX2, and BCX3. The necropsies of tumor-bearing mice and histological examination revealed that 100% of the animals exhibited lung metastases within 50 days for BCX1 (50 of 50) and within 80 days for BCX2 and BCX3 (20 of 20). For BCX1, parallel analyses showed lung metastases in NSG-Pro mice (five of five) but not in NSG mice (zero of five). In addition to lungs (Fig. 3, A to C, and fig. S3, B and C), metastases to bone, liver, and intestines were also observed in NSG-Pro mice bearing these three PDX lines (fig. S3, D to G), suggesting that the PRL-humanized mouse model can facilitate the dissemination of ER^+^ cancer cells to diverse sites, as it frequently occurs in patients with ER^+^ breast cancer.

To assess whether established spontaneous metastatic lesions in NSG-Pro mice remained hPRL dependent, we orthotopically implanted BCX1 tumors, grew them for 55 days, and then surgically resected them. Sixty days later, the mice were treated with either vehicle or bromocriptine, which suppresses the release of PRL from the pituitary gland (*35*), or with a potent antibody antagonizing the hPRLR (*36*). Subsequent analysis of metastatic burden in the lungs revealed that blockade of PRL signaling significantly decreased the BCX1 metastatic burden in the lungs in the absence of the primary tumors that had been excised (Fig. 3, D to G). These results reveal that physiological levels of circulating hPRL in the NSG-Pro mouse can support the growth of latent distant metastases of an ER^+^ breast cancer model and provide the first preclinical evidence of efficacy of PRL pathway targeting against disseminated disease. These data demonstrate that the NSG-Pro host strain provides a novel platform for studying the metastatic progression of ER^+^ breast cancer, i.e., the most prevalent cause of mortality in patients with breast cancer. As documented here, the new mouse model will also allow testing of the efficacies of experimental drugs (e.g., PRLR pathway inhibition) and their combinations in therapy-naïve ER^+^ breast cancer in the postsurgical adjuvant setting, on latent distant metastases before progression to clinically manifest disease.

### The NSG-Pro model permits the study of molecular underpinnings of endocrine resistance developed from therapy-naïve PDX

**Fig. 4.**
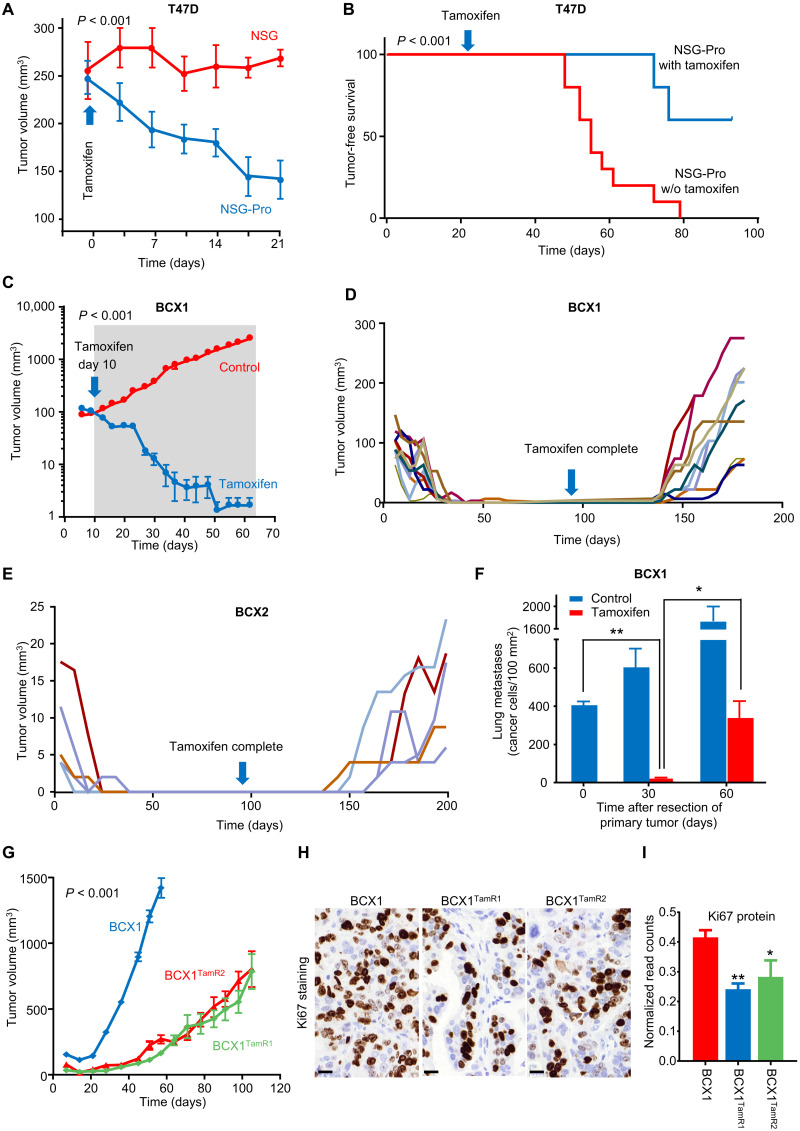
Tamoxifen-induced regression of human breast cancer xenograft models in NSG-Pro mice. (**A**) Responses to continuous tamoxifen of T47D xenograft tumors grown to equal size in NSG-Pro versus NSG mice. (**B**) Kaplan-Meier survival curves of continuously tamoxifen-treated NSG-Pro versus NSG mice xenografted with T47D cells (5 × 10^6^ cells) and monitored for tumor emergence. (**C**) BCX1 PDX tumor size monitored in tamoxifen-treated versus controls in NSG-Pro mice. Shaded area represents duration of tamoxifen treatment. (**D**) Development of tamoxifen-resistant BCX1 tumors in NSG-Pro mice after initial tumor regression in response to 90-day tamoxifen treatment. (**E**) Development of tamoxifen-resistant BCX2 tumors in NSG-Pro mice after initial tumor regression in response to 90-day tamoxifen treatment. (**F**) Reduction of BCX1 lung metastases in NSG-Pro mice after surgical removal of primary tumors in response to 30 days of continuous tamoxifen treatment followed by emergence of tamoxifen-resistant lesions by day 60. (**G**) Growth curve comparison in NSG-Pro mice of BCX1 parental tumors grown under estrogenized conditions versus tamoxifen-resistant BCX1^TamR1^ and BCX1^TamR2^ tumors grown during continuous tamoxifen exposure. (**H**) Representative immunohistochemistry of proliferation marker Ki67 (brown) in BCX1 tumors and tamoxifen-resistant BCX1^TamR1^ and BCX1^TamR2^ tumors grown in NSG-Pro mice. (**I**) Quantification of proliferation marker Ki67 levels in BCX1 and tamoxifen-resistant BCX1^TamR1^ and BCX1^TamR2^ tumors grown in NSG-Pro mice. *P < 0.05; **P < 0.01. Scale bars, 20 μm.

Another critically important challenge for ER^+^ PDX modeling is the failure to establish therapy-naïve, estrogen-dependent in vivo models that later acquire endocrine resistance. We hypothesized that this failure is attributable to the lack of hPRLR-dependent estrogen signaling in ER^+^ tumors grown in conventional mouse strains. For example, tamoxifen frequently regresses ER^+^ breast tumors in premenopausal patients (*37*, *38*), but the drug has historically been unable to regress ER^+^ human breast cancer xenografts in mice, despite decelerating their growth (*39*–*41*). ER^+^ T47D tumors grown in the NSG-Pro recipients underwent a significant regression in response to tamoxifen in contrast to matched tumors of similar size grown in conventional NSG mice (Fig. 4A). The tamoxifen-induced reduction in tumor burden significantly prolonged survival of NSG-Pro subjects (Fig. 4B). The findings demonstrate that physiological levels of hPRL in the NSG-Pro mouse model are critical for accurately recapitulating the therapeutic effects of hormonal therapy, as they are being observed in patients with ER^+^ breast cancer. Consistent with this notion, complete regression of therapy-naïve BCX1 and BCX2 PDX tumors was observed for the duration of tamoxifen treatment and beyond (Fig. 4, C to E). However, tamoxifen-resistant tumors eventually emerged from minimal residual disease within the tumor beds in both PDX models after a period of discontinued tamoxifen treatment (Fig. 4, D and E), recapitulating another important phenomenon associated with the clinical progression of aggressive ER^+^ breast cancer. After surgical removal of primary BCX1 tumors from the mice, lung metastatic lesions remained tamoxifen sensitive and initially regressed almost completely but eventually regrew in the presence of tamoxifen (Fig. 4F). The tamoxifen-resistant PDX lines BCX1^TamR1^ and BCX1^TamR2^ retained the ability to resist continued tamoxifen exposure in subsequent passages (Fig. 4G), albeit displaying slower growth rates as supported by reduced Ki67 levels (Fig. 4, H and I). These results demonstrate that the NSG-Pro host enables the experimental modeling and studies of the development of clinically relevant endocrine resistance, raising the possibility of insights from PDX analyses in NSG-Pro mice that will benefit patients who later experience endocrine-resistant recurrence.

### NSG-Pro mice as preclinical models for prediction of mechanisms of acquired resistance

To expand upon the basic tumor characteristics of BCX1 and to identify molecular targets that might be useful for treating a hypothetical recurrence in this patient, we performed whole-genome sequencing (WGS) of patient blood DNA and DNA from therapy-naïve BCX1 parental tumors and two tamoxifen-resistant derivative lines, BCX1^TamR1^ and BCX1^TamR2^. The WGS analysis identified multiple putative somatic driver mutations and copy number variants (CNVs) that were present in the therapy-naïve parental PDX model and tamoxifen-resistant sublines as well as somatic mutations and CNV that were enriched or lost in BCX1^TamR1^ and BCX1^TamR2^ compared to the parental BCX1 (table S5). The commonly amplified 8q24 region containing the *MYC* locus was detected in all three PDX models BCX1, BCX1^TamR1^, and BCX1^TamR2^ (table S6), whereas 16,508 and 15,286 CNV (>10 kb) were detected in BCX1^TamR1^ and BCX1^TamR2^ compared to BCX1, respectively (table S7). At the molecular level, a damaging missense *TP53* mutation (p.P151T) (*42*) was detected with 100% variant allele frequency (VAF) in BCX1, BCX1^TamR1^, and BCX1^TamR2^. Likewise, a stop-gain mutation affecting both major splice variants of the Ras inhibitor *NF1* (p.Q1399*;p.Q1378*) (*43*) was detected with 100% VAF in all three BCX1 PDX lines.

**Fig. 5.**
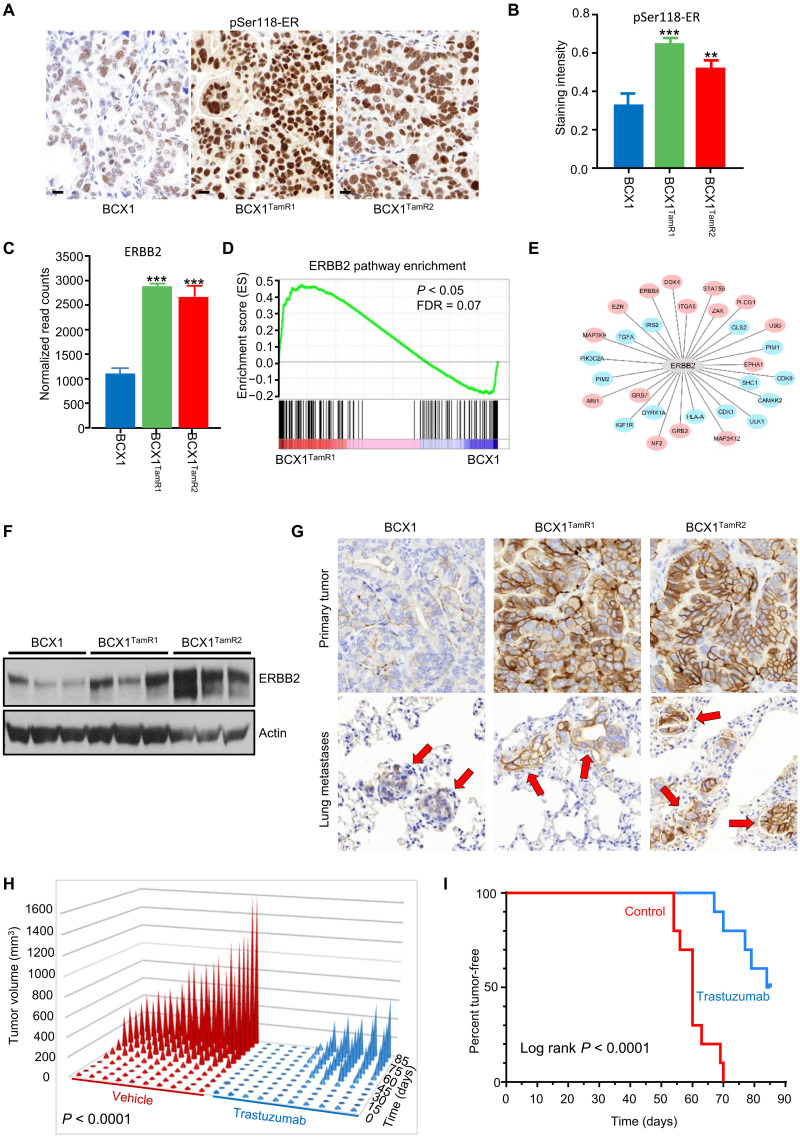
ERBB2 protein up-regulation without gene amplification as tamoxifen escape pathway in ER^+^ PDX model in NSG-Pro mice. (**A**) Representative immunohistochemistry of phosphoSer^118^ of estrogen receptor-α (pSer-ER; brown) in BCX1, BCX1^TamR1^, or BCX1^TamR2^ tumors. (**B**) Quantification of pSer-ER levels in BCX1, BCX1^TamR1^, or BCX1^TamR2^ tumors. (**C**) Transcript levels of human *ERBB2* in BCX1, BCX1^TamR1^, or BCX1^TamR2^ tumors. (**D**) Enrichment score plot of ERBB2 pathway gene transcripts in BCX1^TamR1^ versus BCX1 tumors. (**E**) ERBB2 pathway plot of differentially modulated transcripts in BCX1^TamR1^ versus BCX1 tumors. (**F**) Western blot of ERBB2 protein expression in BCX1, BCX1^TamR1^, and BCX1^TamR2^ tumors. (**G**) Representative immunohistochemistry of ERBB2 expression (brown) in BCX1, BCX1^TamR1^, or BCX1^TamR2^ orthotopic tumors and lung metastases (red arrows). (**H**) Responsiveness of BCX1^TamR1^ tumors to trastuzumab during 91 days of treatment. (**I**) Kaplan-Meier plot of tumor-free survival in BCX1^TamR1^ tumor implanted mice treated with or without trastuzumab. **P < 0.01; ***P < 0.001. Scale bars, 20 μm.

The NF1 mutation observed in the BCX1 tumor increases growth factor Ras signaling (*44*), suggesting that receptor tyrosine kinase (RTK) signaling may represent an early escape pathway in the tamoxifen-resistant sublines BCX1^TamR1^ and BCX1^TamR2^. Tamoxifen-resistant lines BCX1^TamR1^ and BCX1^TamR2^ displayed hyperphosphorylation of ER on serine-118 (Fig. 5, A and B), a molecular mechanism implicated in ligand-independent activation of ER by compensatory growth factor and RTK escape pathways (*45*). Consistent with this mechanism, elevated phosphorylated extracellular signal–regulated kinase levels were observed in tamoxifen-resistant lines (fig. S4, A and B). Collectively, these findings demonstrate that the NSG-Pro mouse recapitulates key aspects of the clinically relevant acquired therapy resistance and metastatic progression of therapy-naïve ER^+^ breast cancer, providing a novel test bed for developing new therapies to avert or control recurrent endocrine-resistant metastatic disease.

To explore this strategy, we examined the RNA-seq data for actionable targets and downstream gene networks that were specifically altered in BCX1^TamR1^ and BCX1^TamR2^ compared to the parental BCX1. This analysis revealed a significant up-regulation of *ERBB2* transcripts and enrichment of ERBB2-modulated transcripts (Fig. 5, C to E) and ERBB2 protein by Western blot and immunohistochemistry (Fig. 5, F and G) in BCX1^TamR1^ and BCX1^TamR2^ compared to BCX1. The ERBB2 protein was also up-regulated in distant metastases of the two tamoxifen-resistant lines (Fig. 5G). WGS analysis or fluorescence in situ hybridization (FISH) analysis in a Clinical Laboratory Improvement Amendments (CLIA)–certified cytogenetics laboratory did not detect *ERBB2* gene amplification in the primary patient tumor, nor was it detected by WGS or FISH in BCX1, BCX1^TamR1^, or BCX1^TamR2^ PDX tumors (fig. S4C), indicating that ERBB2 protein expression is up-regulated in BCX1^TamR1^ and BCX1^TamR2^ by alternative mechanism(s). Consistent with ERBB2-mediated tamoxifen resistance, trastuzumab effectively suppressed the growth of BCX1^TamR1^ tumors and prolonged the tumor-free survival (Fig. 5, H and I), while the parental BCX1 tumors were unresponsive to trastuzumab (fig. S4D). Four of 10 trastuzumab-treated BCX1^TamR1^ tumors did not recur during 90 days of continuous administration of the drug, while delayed growth was observed in the remaining six BCX1^TamR1^ tumors in trastuzumab-treated mice (Fig. 5, H and I). Given that tamoxifen resistance modeled in NSG-Pro mice mimics clinical resistance in patients, the unraveling of an early ERBB2 escape mechanism in PDX tumors might be used for precision-guided therapy of recurrent disease in the patient that cannot be readily predicted simply on the basis of ERBB2 protein levels or *ERBB2* amplification status in the patient’s primary tumor. Standard histological analyses of ER, PR, ERBB2, and Ki67 proteins used in the clinic subtyped the estrogen-dependent BCX1 and BCX2 PDX tumors as luminal B. However, intrinsic subtype classification based on RNA-seq (*46*) unexpectedly classified BCX1 as ERBB2-enriched while confirming BCX2 as luminal B. The absence of *ERBB2* gene amplification and the unresponsiveness to trastuzumab of the tamoxifen-sensitive BCX1 PDX tumors support the protein-based pathology classification. Future work will investigate the conflicting ERBB2-enriched intrinsic molecular subtype of BCX1 and its relevance for subsequent ERBB2-mediated endocrine resistance. Further attesting to utility of the NSG-Pro mouse for breast cancer therapy response prediction, parallel analyses of a separate PDX model developed in NSG-Pro mice, BCX117, revealed resistance to paclitaxel but responsiveness to carboplatin, mimicking disease resistance and responsiveness observed in the patient (fig. S4E). Collectively, these observations on drug responsiveness highlight the significant advantages of human breast cancer tested in the NSG-Pro mouse model, allowing preclinical validation of rationally tailored therapies based on molecular profiling of tumors.

### The new NSG-Pro model is a preclinical platform for developing patient-based precision therapies

Developing precision therapies using ER^+^ PDX models has been markedly limited by the abovementioned challenges in establishing therapy-naïve ER^+^ PDX models in mice that metastasize and eventually develop therapy resistance. Hence, we explored whether the NSG-Pro mouse would be more amenable to developing precision-guided therapies for patients with ER^+^ breast cancer. For example, the BCX1 PDX model that we developed in the NSG-Pro mouse originated from a premenopausal patient with an ER^+^/PR^+^/ERRB2^−^ breast tumor; this patient’s tumor was subjected to the standard-of-care diagnostics, which did not include genomic and transcriptomic analysis. To provide proof-of-principle evidence that the NSG-Pro mouse can be used to identify actionable targets that may guide patient care in real time, we sought to identify additional targetable mechanisms that might drive therapy-resistant disease upon eventual recurrence in this patient.

**Fig. 6.**
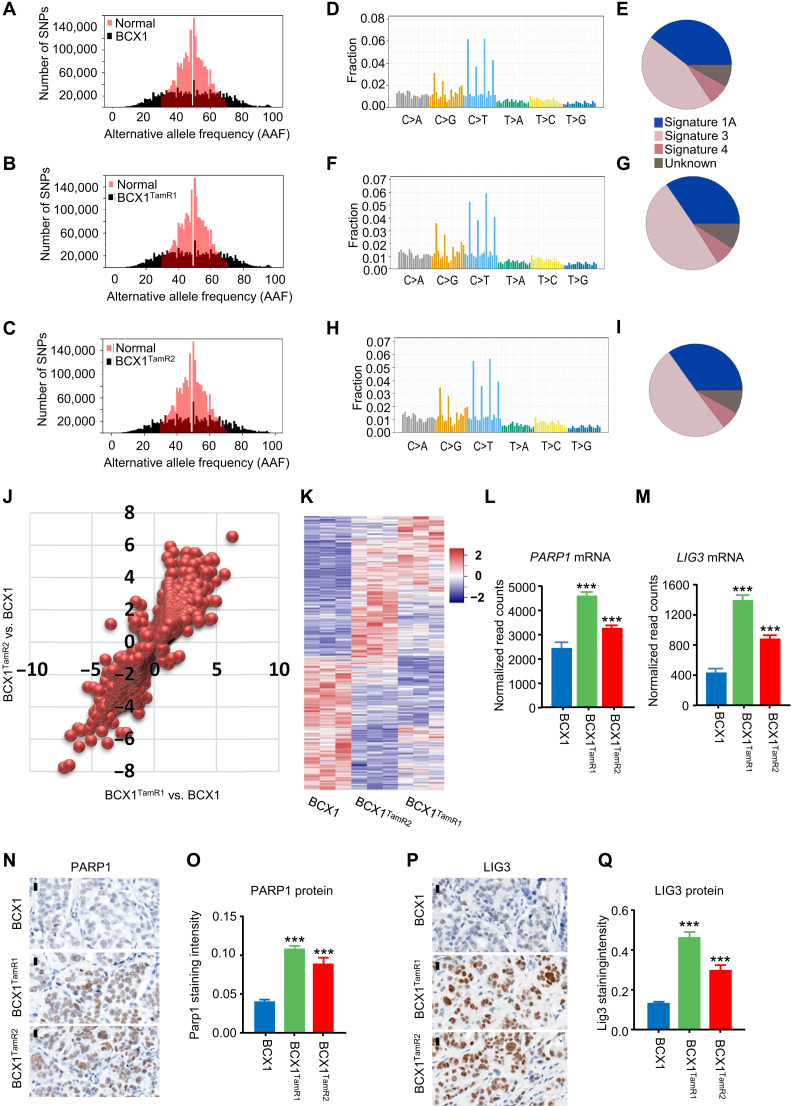
ER^+^ PDX breast cancer model with genomic instability. (**A**) Alternative allele frequency plot of normal blood DNA of patient versus DNA of BCX1 PDX tumors. SNP, single-nucleotide polymorphism. (**B**) Alternative allele frequency plot of normal blood DNA of patient versus DNA of BCX1^TamR1^ PDX tumors. (**C**) Alternative allele frequency plot of normal blood DNA of patient versus DNA of BCX1^TamR2^ PDX tumors. (**D**) Mutation frequency plot of normal blood DNA of patient versus DNA of BCX1 PDX tumors. (**E**) Gene mutation signature of BCX1 PDX tumors. (**F**) Mutation frequency plot of normal blood DNA of patient versus DNA of BCX1^TamR1^ PDX tumors. (**G**) Gene mutation signature of BCX1^TamR1^ PDX tumors. (**H**) Mutation frequency plot of normal blood DNA of patient versus DNA of BCX1^TamR2^ PDX tumors. (**I**) Gene mutation signature of BCX1^TamR2^ PDX tumors. (**J**) Scatter plot of modulated human-specific gene transcripts in BCX1^TamR1^ and BCX1^TamR2^ versus BCX1 tumors. (**K**) Heatmap of most significant up- or down-modulated human gene transcripts in BCX1^TamR1^ and BCX1^TamR2^ versus BCX1 tumors. (**L**) Bar graph of human *PARP1* mRNA levels in BCX1^TamR1^ and BCX1^TamR2^ versus BCX1 tumors. (**M**) Bar graph of human *LIG3* mRNA levels in BCX1^TamR1^ and BCX1^TamR2^ versus BCX1 tumors. (**N**) Representative immunohistochemistry of PARP1 protein (brown) in BCX1^TamR1^ and BCX1^TamR2^ versus BCX1 tumors. (**O**) Quantification of PARP1 protein expression in BCX1^TamR1^ and BCX1^TamR2^ versus BCX1 tumors. (**P**) Representative immunohistochemistry of LIG3 protein (brown) in BCX1^TamR1^ and BCX1^TamR2^ versus BCX1 tumors. (**Q**) Quantification of LIG3 protein expression in BCX1^TamR1^ and BCX1^TamR2^ versus BCX1 tumors. ***P < 0.001. Scale bars, 20 μm.

Global analysis of loss of heterozygosity (LOH) revealed that BCX1 (and, to an even greater extent, BCX1^TamR1^ and BCX1^TamR2^) exhibited a high degree of functional aneuploidy (Fig. 6, A to C) as defined by the extent of LOH across the genome (*47*). Mutational signature analysis revealed a predominant pattern of somatic mutations that was indicative of BRCA1/2 pathway deficiencies in homology-directed repair (HDR; signature 3) (*48*), which was present in BCX1 and slightly more enriched in BCX1^TamR1^ and BCX1^TamR2^ (Fig. 6, D to I). In addition to the TP53 and NF1 driver mutations, somatic mutations within the HDR pathway were enriched in BCX1^TamR1^ and BCX1^TamR2^ compared to BCX1, including *TOP3A* (p.P706A; >50-fold enriched VAF) and *BRCA2* (p.K1568N; 1.4-fold enriched VAF; table S5). Collectively, these findings suggest that parental BCX1 cells harbored genetic alterations in the HDR pathway and that these alterations were selectively enriched in the treatment-resistant BCX1^TamR1^ and BCX1^TamR2^ PDX lines. The BRCA2-p.K1568N mutation detected in BCX1 tumors is currently considered of unlikely significance for familial breast cancer risk as a somatic mutation (*49*). However, future work will interrogate its significance as a somatic mutation during cancer progression and as a mediator of therapy resistance.

Detection of HDR pathway disruption in ER^+^ PDX models is clinically relevant to tailoring a patient-specific treatment plan to include PARP inhibitors that are highly effective in patients with HDR deficiencies (*50*, *51*). PARP inhibitors are prescribed on the basis of the presence of BRCA1 or BRCA2 mutations that are predicted to disrupt HDR; however, PDX-derived pathway data, to our knowledge, have not previously been explored. Likewise, it is unclear what role physiological levels of hPRL play in the detection of HDR pathway biomarkers and sensitivity to PARP inhibitors. To address these questions, we examined RNA-seq data from BCX1 tumors grown in NSG-Pro or conventional NSG mice (Fig. 2, C to H) to determine whether HDR pathway members were modulated by hPRL. We observed a significant enrichment of HDR pathway members (including up-regulation of *BRCA1*, *BRCA2*, *RAD50*, and *RAD51AP1*) that were DE in BCX1 tumors grown in NSG-Pro mice compared to conventional NSG mice (FDR-adjusted *P* < 0.05; table S3), suggesting that the hPRL/PRLR signaling axis modulates HDR phenotypes in ER^+^ breast cancer PDX. Next, we used RNA-seq on the BCX1, BCX1^TamR1^, and BCX1^TamR2^ PDX lines in NSG-Pro mice to assess whether HDR and other DNA repair pathways were further perturbed during acquired tamoxifen resistance. Of the 3986 DE genes in BCX1^TamR1^ and BCX1^TamR2^ compared to BCX1 (Fig. 6, J and K; fig. S4F; and tables S8 and S9), *BRCA2* expression was reduced (FDR-adjusted *P* < 0.01; tables S8 and S9), and DNA repair genes in the alternative end-joining pathway, *PARP1* and *LIG3*, were up-regulated at the mRNA (Fig. 6, L and M) and protein levels (Fig. 6, N to Q). Collectively, the data suggested that the patient’s primary tumor, from which the BCX1 PDX model was derived, likely had intrinsic genomic instability due to HDR deficiencies that remained undiagnosed by standard techniques, demonstrating the value of the NSG-Pro mouse in developing clinically relevant PDX models for directing patient care.

**Fig. 7.**
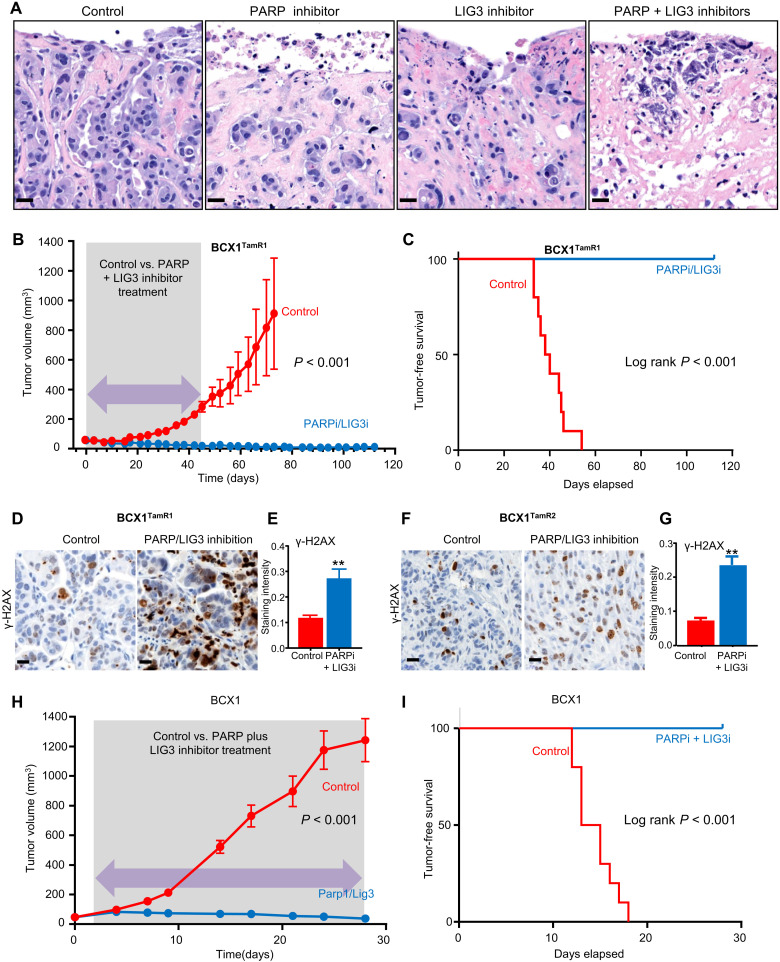
Responsiveness of HDR-deficient primary and tamoxifen-resistant ER^+^ breast cancer models to targeted inhibition of PARP and LIG3. (**A**) Histology of tamoxifen-resistant BCX1^TamR1^ tumor explants exposed ex vivo to control medium, PARP inhibitor talazoparib, LIG3 inhibitor L67, or the combination of talazoparib and L67 for 7 days. (**B**) BCX1^TamR1^ tumor growth curves in NSG-Pro mice bearing slow-release tamoxifen pellets and treated with either vehicle or the combination of PARP inhibitor (PARPi) talazoparib and LIG3 inhibitor (LIG3i) L67 for 42 days (shaded interval). Mice were followed for tumor development up to 117 days. (**C**) Kaplan-Meier survival analysis of tumor-free status in NSG-Pro mice bearing slow-release tamoxifen pellets implanted with BCX1^TamR1^ tumors and treated with either vehicle or the combination of PARP inhibitor talazoparib and LIG3 inhibitor L67 for 42 days. (**D**) Representative immunohistochemistry of γ-H2AX (brown) in BCX1^TamR1^ tumors exposed for 24 hours to PARP/LIG3 inhibitor combination or vehicle (control). (**E**) Quantification of γ-H2AX levels in BCX1^TamR1^ tumors exposed for 24 hours to PARP/LIG3 inhibitor combination or vehicle (control). (**F**) Representative immunohistochemistry of γ-H2AX (brown) in BCX1^TamR2^ tumors exposed for 24 hours to PARP/LIG3 inhibitor combination or vehicle (control). (**G**) Quantification of γ-H2AX levels in BCX1^TamR2^ tumors exposed for 24 hours to PARP/LIG3 inhibitor combination or vehicle (control). (**H**) BCX1 tumor growth curves in NSG-Pro mice bearing slow-release tamoxifen pellets and treated with either vehicle or the combination of PARP inhibitor talazoparib and LIG3 inhibitor L67 for 28 days. (**I**) Kaplan-Meier survival analysis of tumor-free status in NSG-Pro mice bearing slow-release tamoxifen pellets implanted with BCX1 tumors and treated with either vehicle or the combination of PARP inhibitor talazoparib and LIG3 inhibitor L67. **P < 0.01; ***P < 0.001. Scale bars, 20 μm.

PARP1 and LIG3 are actionable targets whose activities are frequently up-regulated in response to the accumulation of single-strand breaks and double-strand breaks in DNA that accumulate from HDR deficiency (*52*, *53*). To assess whether the knowledge gained from the WGS and RNA-seq analysis in our study could be leveraged to develop precision-guided therapies for the patient in case of future therapy-resistant recurrence, we assessed the significance of inhibitors of PARP (talazoparib) and LIG3 (L67) to suppress growth of tamoxifen-resistant BCX1 in vivo. First, we exposed ex vivo long-term 3D explant cultures of BCX1^TamR1^ tumors harvested from NSG-Pro mice for 7 days to talazoparib and L67, alone or in combination, revealing individual drug efficacy as well as an additive effect when combined (Fig. 7A). Second, transient 42-day combination therapy of talazoparib and L67 in vivo was remarkably efficacious in suppressing the long-term growth of the tamoxifen-resistant BCX1^TamR1^ tumors, with complete responses observed for all animals over the 80-day follow-up period after completion of therapy (Fig. 7, B and C). This treatment was well tolerated by animals without measurable weight loss (fig. S4G). Third, short-term in vivo treatment showed that both tamoxifen-resistant BCX1^TamR1^ and BCX1^TamR2^ were responsive to this drug combination within 24 hours using γ-H2AX up-regulation as an early drug response readout (Fig. 7, D to G). Last, we demonstrated that the parental BCX1 tumors were also sensitive to combined PARP and LIG3 inhibitor treatment in vivo, consistent with the identified HDR pathway aberrations (Fig. 7, H and I). Together, the results from this study clearly demonstrate that the NSG-Pro mouse model holds great potential for predictive analysis that ultimately could be used to enhance patient care in cases with tumor recurrence following endocrine therapy.

## DISCUSSION

Orthotopic breast cancer PDX models have existed for nearly half a century (*54*), yet they have been plagued by the inability to capture a diverse set of all breast cancer subtypes. Most notably, therapy-naïve primary luminal or ER^+^ breast tumors have historically yielded engraftment rates below 5% compared to much greater take rates of the ER^−^ subtypes. Here, we describe the development and characterization of the PRL-humanized NSG-Pro mouse model, which is the first PDX recipient strain to support the engraftment, growth, metastatic progression, and the development of resistance to clinically relevant therapies across diverse breast cancer subtypes, in particular therapy-naïve ER^+^ tumors. We demonstrate that humanization of PRL in the NSG-Pro mouse model resolves the critical incompatibility between mPRL and hPRLR and restores cooperative ER and PRL signaling in engrafted human breast cancer cells. By rescuing the requisite hPRL/hPRLR signaling axis, we observed a marked increase in the engraftment rates of ER^+^ PDX to more than 40%, while maintaining a comparably high engraftment of ER^−^ tumors. Histological features and mutational profiles of breast cancer–relevant genes were generally concordant between PDX tumors and the primary tumors (Fig. 2, fig. S2, and table S10). In support of emerging evidence for prometastatic roles for PRL (*26*, *27*, *55*), the NSG-Pro model uniquely recapitulate the progression of therapy-naïve, estrogen-dependent primary ER^+^ tumors to systemic disease. In addition to spontaneous lung metastases, ER^+^ PDX tumors grown in these mice retain the ability metastasize to the bone and liver, i.e., other common sites of clinical metastases of luminal breast cancers (*56*). In proof-of-principle experiments using an array of genomic, transcriptomic, and pharmacological analyses, we demonstrated that the NSG-Pro model represents a superior preclinical platform for identifying actionable targets and precision medicine strategies to overcome therapy resistance to treat metastatic ER^+^ disease, which is the major cause of breast cancer patient mortality.

NSG-Pro is a humanized knockin model and provides physiological levels of hPRL under the control of the endogenous mouse *Prl* gene and lacks mPRL, which is a partial hPRLR antagonist (*29*). The physiological nature of the novel NSG-Pro model is important because patients with breast cancer are rarely hyperprolactinemic (*32*). We demonstrated that adjuvant pharmacological suppression of circulating hPRL or inhibition of hPRLR significantly inhibited growth of established latent metastatic lung lesions of a therapy-naïve ER^+^ breast cancer PDX model. The physiological hPRL levels in the NSG-Pro mouse were critical for growth, metastasis, and tamoxifen responsiveness of engrafted ER^+^ breast cancer cell lines and PDX. Intriguingly, it has been a longstanding conundrum in the field that ER^+^ breast cancer xenografts grown in mice do not regress in response to tamoxifen (*39*–*41*), a phenomenon that is otherwise commonly being observed in patient tumors exposed to neoadjuvant tamoxifen (*37*). We demonstrated that tamoxifen induced a marked regression of T47D xenograft tumors in the NSG-Pro mice but not in conventional NSG controls. In addition, three of the three therapy-naïve ER^+^ PDX tumor models tested (BCX1, BCX2, and BCX3) also consistently regressed in response to tamoxifen in NSG-Pro mice. Eventually, tamoxifen-resistant tumors emerged in each of these PDX models from a minor pool of residual cancer cells. BCX1 tumors with acquired tamoxifen resistance consistently showed ERBB2 protein up-regulation without gene amplification and became sensitive to trastuzumab. Notably, we found that hPRL was critical for expression of genes within the ER and ERBB2 pathways, both in vitro and in vivo. Thus, NSG-Pro mice may help identify tumors that will default to the ERBB2 escape pathway, allowing patients with these tumors to be tested for benefit of combined targeting of ERBB2 and ER signaling to overcome or prevent endocrine resistance. On the basis of the initial characterization of NSG-Pro, we propose that physiological levels of hPRL restore the endocrine environment adequate for ER^+^ breast cancer biology and drug responsiveness, making NSG-Pro mice more applicable for testing of pharmacological agents against ER^+^ breast cancer than conventional NSG mice.

Using the NSG-Pro mouse, we have established a growing panel of serially transplantable therapy-naïve ER^+^ breast cancer PDX models that spontaneously metastasize to vital organs, including lungs, with 100% mortality. This is important because agents can now be effectively tested for efficacy in the adjuvant subclinical or latent metastatic setting in NSG-Pro mice after surgical removal of primary tumors. While encouraging progress is being made with patient-derived tumor organoids cultured in plastic for personalized cancer medicine (*57*–*59*), the known modulatory effects of tumor microenvironment factors on therapeutic responses of cancer (*60*, *61*) remain a limitation of stroma-free tumor organoid cultures. Analyses of mechanisms underlying drug resistance as it develops in vivo in therapy-naïve distant metastases of ER^+^ breast cancers cannot be assessed in organoid cultures but can now be analyzed in NSG-Pro mice. Because clinical recurrence of latent metastatic ER^+^ breast cancer typically takes years, there is, in fact, sufficient time to gain predictive insights from drug testing of PDX models. For instance, should breast cancer recur in the patient from which PDX-BCX1 and tamoxifen-resistant sublines were derived; our progress in NSG-Pro mice predicts that PARP plus LIG3 inhibitors would be effective.

Germline *BRCA1/2* mutations are typically associated with increased risk of TNBC (*62*). Somatic mutations in *BRCA1/2* are uncommon in primary ER^+^ breast cancer (*6*, *63*). Using the new NSG-Pro recipient strain with ER^+^ PDX models, we uncovered evidence of actionable somatic *BRCA1/2* pathway disruption in tamoxifen-resistant, isogenic PDX tumor sublines. In an ER^+^ PDX model established in NSG-Pro mice, we identified the somatic *BRCA2*-K1568N mutation, a genetic alteration currently of uncertain significance (*49*). Nonetheless, because WGS of the tumor uncovered a somatic mutational signature that is broadly indicative of *BRCA1/2* pathway disruption (*48*), this prompted us to explore DNA repair inhibitors, revealing remarkable efficacy of combined pharmacologic targeting of PARP1 and LIG3 in both therapy-naïve parental tumors and tamoxifen-resistant derivatives. Access to sufficient PDX tumor tissue from mice allowed us to explore ex vivo the efficacy of a combination therapy that was validated in vivo.

A major strength of the NSG-Pro mouse as a host for breast cancer PDX-development and drug response testing is the rescue of prolactin receptor (PRLR) signaling, which modulated the major therapy-targeted ESR1 and ERBB2 pathways based on molecular profiling of ER^+^ PDX tumors grown in NSG-Pro versus NSG mice. However, the extensive molecular characterization of the initial ER^+^ PDX models reported here can be further strengthened by deeper analyses of the molecular concordance between each therapy-naïve patient tumor and its derived therapy-naïve PDX tumor line at the genomic, transcriptomic, and proteomic levels. Preserving adequate fresh-frozen patient tumor tissue that is representative of that implanted into mice will allow for more accurate comparisons of nucleic acid sequence data and proteomic profiles. Additional comparisons of drug responsiveness of therapy-resistant sublines of the PDX tumors with that of matched, recurring patient tumors will be possible once recurrences occur. It will be interesting to determine whether the propensity of individual PDX tumor lines to spontaneously metastasize in NSG-Pro mice has prognostic significance and correlates with future development of distant metastases in the patient. While the data presented in this report provides compelling benefits of rescuing hPRL signaling in mice for PDX modeling of ER^+^ breast cancer, it is likely that correction of additional key molecular incompatibilities between mice and humans will be needed to further harmonize biology and drug responsiveness of patient tumors and PDX tumor lines.

In summary, the novel NSG-Pro model holds great promise as a test bed for developing precision medicine strategies for breast cancer, including the common but difficult-to-study luminal breast cancers. Beyond the first proof-of-principle efficacy of adjuvant pharmacological hPRL pathway targeting on residual lung metastases of human ER^+^ breast cancer documented here, future studies will determine the broader usefulness of the NSG-Pro mouse in development and preclinical assessment of novel adjuvant therapies for disseminated disease. The NSG-Pro mouse model will yield novel insights into tumor biology, metastatic progression, mechanisms of therapy resistance, and therapeutic approaches to target metastatic disease and endocrine resistance.

## MATERIALS AND METHODS

### Experimental design: Development of the NSG-Pro mouse model

A bacterial artificial chromosome (BAC) clone encompassing the murine *prl* gene locus was isolated from the genomic library created from the CJ7 cell line derived from 129/Sv mouse line (cat. no. 96040H, Invitrogen, Carlsbad, CA). A 5.2-kb Apa I/Pst I fragment harboring the 5′ region upstream of exon 1 was isolated and cloned into pBlueScript. The 5′ untranslated region (5′UTR) was generated by annealing two oligos: 5′-GATGAGAAAGCTGTGGTTCTCTCAGGCCATCTTGGAGAAGTGTGTTCCCAGCAGTCACC-3′ and 5′-GGTGACTGCTGGGAACACACTTCTCCAAGATGGCCTGAGAGAACCACAGCTTTCTCATCT-3 containing a Pst I site and a blunt end at the 5′ end and 3′ end, respectively. The *hPRL* complementary DNA (cDNA) was amplified from human brain total RNA (BioChain, Hayward, CA) by reverse transcription reaction (Bio-Rad, Hercules, CA) and polymerase chain reaction (PCR) using Pfx polymerase (Invitrogen, Carlsbad, CA) and a primer pair: 5′-ATGAACATCAAAGGATCGCC-3′ and 5′-ATAGTTTAGCGGCCGCGTCGACAAGCTTTTAGCAGTTGTTGTTGTGG-3′, introducing Hind III, Sal I, and Not I sites on the 3′ end of the amplified product. The 5′UTR and hPRL cDNA were inserted downstream of 5.2-kb Apa I/Pst I to create the 5′ARM-UTR-hPRL. A 2.1-kb fragment harboring intron 1 through part of intron 2 was amplified from the BAC clone by PCR using Pfx polymerase and a primer pair: 5′-CTATCTCGAGGTATGTTCTGTAGCTTAGTGAC-3′ and 5′-ATAAGCGGCCGCGAAGGTCGCTTCACTTTTGC-3′, introducing Xho I and Not I sites on the 5′ end and 3′ end, respectively, which was inserted into the pLoxP targeting vector at the Xho I and Not I sites, downstream of the neomycin selection cassette (neo) and *lox*P site. Subsequently, 5′ARM-UTR-*hPRL* was inserted upstream of the *lox*P site and Neo cassette at Kpn I and Sal I in pLoxP. The targeting vector was fully sequenced and was linearized using Not I. The generation of *hPRL* knockin mice, including embryonic stem cell (ES) targeting, screening, and expansion, blastocyst injections, and germline transmission, was performed as a subcontract by Cell & Molecular Technologies Inc. (Phillipsburg, NJ 08865). Removal of the floxed neomycin cassette was achieved by breeding with a germline CRE-expressing mouse as described (*33*).

### Cell lines

Human T47D, MCF7, and ZR75.1 (American Type Culture Collection, Manassas, VA) were maintained as monolayer cultures in RPMI 1640 medium (10-040-CV; Corning) supplemented with 10% fetal bovine serum (FBS) (26140-079; Gibco) and 1% penicillin-streptomycin (P/S) solution (P4333; Corning) at 37°C, 5% CO_2_, and 95% relative humidity. Murine 32D cells transfected with murine PRL receptor cDNA were maintained in the same basal medium supplemented with 1 nM murine PRL.

### 3D spheroid assay

To grow spheroids, T47D cells were seeded into ultralow attachment 24-well plates (3473; Corning, Tewksbury, MA) at 100,000 cells/ml in RPMI 1640 medium (R8755; MilliporeSigma, Burlington, MA) supplemented with 0.5% methylcellulose (M0387; MilliporeSigma), 1% charcoal-stripped FBS (12676029; Gibco, Waltham, MA), 10 nM 17β-estradiol (E2578; MilliporeSigma), and 10 nM progesterone (P8783; MilliporeSigma) and increasing concentrations of recombinant hPRL (100-07; PeproTech, Rocky Hill, NJ) or mPRL (315-16; PeproTech). After 7 days of growth, the cell suspensions were harvested into 1.5-ml microcentrifuge tubes, and the remaining contents of the wells were collected with 0.5 ml of Dulbecco’s phosphate-buffered saline (DPBS) (23-031-CV; Corning). The cells were spun at 1200*g* for 4 min, the supernatant was aspirated, and the cell pellets were resuspended in 100 μl of cell counting solution prepared by diluting 1-part Cell Counting Kit-8 (CK04; Dojindo Molecular Technologies, Rockville, Maryland) reagent with 9-parts DPBS. The cells were incubated at 37°C for 1 hour and transferred to a 96-well plate. The relative cell number was assessed by measuring absorbance at 495 nm and is reported as the fold change in response to treatment relative to control conditions. Triplicate experiments were performed on two separate occasions.

### Cell proliferation assay

The mouse lymphoblast cell line 32D was stably transfected with a plasmid carrying mPRL receptor cDNA using electroporation. Stable clones were pooled and grown in RPMI 1640 medium supplemented with puromycin (0.5 μg/ml), 10% FBS, 2 mM l-glutamine, 1 nM mPRL, and P/S (50 IU/ml and 50 μg/ml, respectively). For proliferation assay, cells were lactogen-starved for 16 hours in RPMI 1640 supplemented with 10% HS (horse serum), 2 mM l-Glu, and 1% P/S for 16 hours and incubated with various concentrations of either hPRL or mPRL for 48 hours. MTS assays were performed as directed by the manufacturer (Promega, Madison, WI), and absorbance [optical density (OD) at 490 nm] was recorded using a Bio-Rad Microplate Reader 680 (Bio-Rad, Hercules, CA). Absorbance recordings were normalized to no PRL control cells and graphed as relative cell density (fold increase; ±SEM) using SigmaPlot (Systat Software Inc., San Jose, CA). The data were derived from six experiments.

### hPRL enzyme-linked immunosorbent assays

Serum from NSG and NSG-Pro mice were collected, and hPRL levels in mouse serum were quantified using an enzyme-linked immunosorbent assay kit (R&D Systems, Minneapolis, MN, USA) following the manufacturer’s instructions. Results were presented as means ± SD.

### Engraftment of PDXs of breast cancer in NSG-Pro mice

Fresh tumor tissue samples were collected under Institutional Review Board–approved protocols from consenting patients undergoing surgical excision of breast cancer. Surgical specimens were received within an hour of excision. Samples were placed into a petri dish with ice-cold RPMI 1640 containing P/S. Sterile forceps and scissors were used to trim away extraneous fat. A small piece of each tumor specimen was collected for formalin fixation and use in histological analyses. The remaining tumor tissue was prepared for implantation into one to three mice by mincing into approximately 1-mm^3^ tissue fragments using sterile scalpels. NSG-Pro mice were anesthetized using 2% isoflurane and placed on a heating pad, and abdominal hair above nipples of the fourth paired mammary glands were removed by Nair. The skin was disinfected using gauze pads with both iodine and 70% ethanol. A 17β-estradiol pellet [1 mg/90-day slow release; Innovative Research of America (IRA), Sarasota, FL] was implanted subcutaneously using a 10-gauge trocar. A 12-gauge trocar was loaded with tumor fragments and injected orthotopically into one of the fourth paired mammary glands. Each injection site was sealed with Vetbond surgical glue (3M, St. Paul, MN), and antibiotic ointment was applied. The mice were left on the heating pad and monitored until they recovered from anesthesia. During xenograft development, mice were palpated, and tumor growth was measured using caliper twice weekly. Following visible and palpable growth, tumors were dissected from the mammary glands, and fragments were retransplanted into new mice and expanded following the same procedures.

### Development of endocrine-resistant models

Fresh xenograft tumor fragments of BCX1, BCX2, or BCX3 PDX lines were transplanted into fat pads of recipient NSG-Pro mice. Tamoxifen pellets (10 mg/90-day slow release; IRA, Sarasota, FL) were implanted simultaneously. After the initial 90 days, depleted tamoxifen pellets were not initially replaced, allowing surviving residual cancer cells to regrow. On subsequent transplantations, tamoxifen treatment was maintained continuously. Tumor size was monitored weekly using calipers and growth curves were plotted.

### Xenograft studies

All animal studies were performed under approved Institutional Animal Care and Use Committee protocols. MCF7, T47D, and ZR75 xenografting were performed in NSG and NSG-Pro estrogenized female mice (8 to 10 weeks old, 17β-estradiol pellets; 1 mg per 90-day release; IRA, Sarasota, FL, USA). For the tamoxifen study of T47D tumors, control-treated (*n* = 5) or tamoxifen-treated mice (*n* = 5; 10 mg per 90-day release; IRA) were grown and harvested as indicated. Tumor size was monitored twice weekly using calipers, and growth curves were plotted. For PARP/Lig3 inhibitor experiments, fresh xenograft tumor fragments of BCX1 and BCX1^TamR1^ were transplanted orthotopically into mammary fat pads of recipient NSG-Pro mice. When tumors reached a volume of 50 to 100 mm^3^, mice were randomized and treated with either vehicle (five mice) or combination of talazoparib [0.33 mg/kg, intraperitoneal injection, five times a week (Monday to Friday)] and L67 [20 mg/kg, oral gavage, five times a week (Monday to Friday); five mice]. The duration of treatment was 6 weeks for BCX1^TamR1^ study and 4 weeks for BCX1 study. For trastuzumab studies, fresh xenograft tumor fragments of BCX1^TamR1^ or BCX1 were transplanted into fat pads of recipient NSG-Pro mice. When tumors reached a volume of 50 to 100 mm^3^, mice were randomized and treated with either vehicle (five mice) or trastuzumab (20 mg/kg, intraperitoneal injection, once a week; five mice) for a period of 9 weeks.

### Immunohistochemistry and histologic evaluation of tumors

Transplanted tumors were fixed in 10% neutral buffered formalin, paraffin-embedded, and hematoxylin and eosin–stained. Tumors were reviewed by breast pathologist (J.M.J.) and analyzed by immunohistochemistry for expression of protein markers (ERα, DAKO-M7047; PR, DAKO-M3568; ERBB2, DAKO-A0485; Ki67, DAKO-M7240; LIG3, Sigma-Aldrich-HPA006723; PARP1, Sigma-Aldrich-HPA045168; pTyr-STAT5; Epitomics-E208; and human-specific mitochondrial protein, Sigma-Aldrich-MAB1273) with positive and negative controls included.

### Cell pellet immunohistochemistry

T47D cells were treated with 50% serum from NSG or NSG-Pro mice for 1 hour and then fixed in 10% formalin for 20 min. Cell pellets were obtained by centrifuging the fixed cells for 10 min at 800*g*. Cell pellets were resuspended at 45°C in melted HistoGel (Thermo Fisher Scientific, Waltham, MA) at a 1:1 volume ratio, solidified on ice, and further formalin-fixed overnight before processing and embedding into paraffin. Microtome sections were analyzed for pTyr-Stat5 (Epitomics-E208) as previously described (*64*).

### Ex vivo 3D tumor explant cultures of PDX tumors

Long-term explant cultures of breast cancer PDX tissues were generated as previously described (*65*, *66*). Briefly, PDX tissues were minced into approximately 1-mm^3^ fragments in basal medium, transferred onto matrix-covered grids in petri dishes, and cultured at 37°C in a mixture of oxygen, carbon dioxide, and nitrogen (40:5:55) for 7 days in the presence or absence of talazoparib (100 μM) and/or L67 (100 μM). The basal medium was Medium 199 with Earle’s salts (Sigma-Aldrich) containing 10% FBS, G-penicillin (100 IU/ml), streptomycin sulfate (100 μl/ml), and glutamine (100 μg/ml) and supplemented with insulin (0.08 IU/ml; Novo Nordisk), dexamethasone (100 nM; Sigma-Aldrich), PRL (100 nM), and 17β-estradiol (100 nM). The culture medium and agents were changed every other day.

### Efficacy of adjuvant treatment with hPRLR antagonist, bromocriptine, or tamoxifen on established lung metastases

Fresh xenograft tumor fragments of BCX1 were transplanted into mammary fat pads of recipient NSG-Pro mice. Tumors were surgically removed when they reached a volume of approximately 1000 mm^3^. Mice were allowed to recover for 2 weeks. For bromocriptine study, mice were randomized and treated for 14 days with either placebo pellet (10 mice) or bromocriptine pellet (10 mg/60-day slow release; 10 mice). For hPRLR antagonist study, mice were randomized into groups of 10 and treated for 10 days with either mouse immunoglobulin G2a (IgG2a) isotype control (20 mg/kg, intraperitoneal injection, every 7 days; BioXCell clone C1.18.4) or chimeric anti-hPRLR (20 mg/kg, intraperitoneal injection, every 7 days). Chimeric anti-hPRLR was designed by grafting the humanized VH and VL domains of hPRLR-neutralizing monoclonal antibody LFA102 (*67*) onto mouse IgG2a heavy and mouse κ light constant domains. Antibody was expressed in human embryonic kidney 239 cells, purified by protein G Sepharose, and cleared of endotoxin (<5 EU/mg). For tamoxifen experiment, mice were randomized and treated with either placebo pellet (10 mice) or tamoxifen pellet (10 mg/60-day slow release; 10 mice) for 30 or 60 days. For all experiments, mice were euthanized, and lungs were formalin-fixed for quantitative lung metastasis analyses. Lungs were collected, and lung metastases were analyzed by immunohistochemistry for human-specific mitochondrial protein (Sigma-Aldrich, MAB1273).

### Western blot analysis/immunoblotting

Pituitaries from mice that were either wild type, heterozygous, or homozygous for *hPRL* were lysed using T-PER (Tissue Protein Extraction Reagent) (Thermo Fisher Scientific, Grand Island, NY) following the manufacturer’s protocol. Proteins were resolved by SDS–polyacrylamide gel electrophoresis and immunoblotted with polyclonal rabbit anti-PRL antibody (1:1500) as described previously (*29*). PDX tumor tissue from BCX1, BCX1^TamR1^, and BCX^TamR2^ were lysed, and proteins were resolved by SDS–polyacrylamide gel electrophoresis and immunoblotted with rabbit monoclonal anti-HER2 antibody (1:1000; Cell Marque, Rocklin, CA).

### RNA-seq and WGS

Cultured T47D cells were treated with either 2.5 nM murine PRL or 2.5 nM hPRL for 12 hours. RNA was extracted following the manufacturer’s instructions (QIAGEN, Germantown, MD) and subjected to RNA-seq. For all nucleic acid analyses of human xenografts in mice, human-specific nucleic acid analyses were performed to eliminate contribution from rodent stromal cells as described previously (*68*). All RNA-seq analyses include three to five biological replicates (T47D treated with mPRL or hPRL, triplicate; RNA-seq for BCX1, BCX1^TamR1^, and BCX1^TamR2^, triplicate; BCX1 grown in either NSG or NSG-Pro mice, quintuplicate; and BCX2 grown in either NSG or NSG-Pro mice, quadruplicate). Tumor tissues from BCX1, BCX1^TamR1^, and BCX^TamR2^ PDX lines were collected, and both RNA and DNA were extracted. RNA was subjected to RNA-seq, and DNA was subjected to WGS. Tumor tissues of BCX1 or BCX2 grown in either NSG or NSG-Pro mice were collected, and RNA was extracted and subjected to RNA-seq. For RNA-seq, total RNA (4 μg) was poly-A–purified, transcribed, and chemically fragmented using Illumina’s TruSeq RNA library kit per the manufacturer’s protocol. Individual libraries were prepared for each sample, indexed for multiplexing, and then sequenced on an Illumina HiSeq 2500 (Illumina Inc., San Diego, CA). Genome sequence and GTF files were obtained from Ensembl. The RSEM (RNA-Seq by Expectation-maximization) program function “rsem-prepare-reference” (v1.3.0) was used to extract the transcript sequences from the human genome (build GRCh38) (*69*) and to generate Bowtie2 indices (Bowtie2 v2.2.8) (*70*) followed by read alignment using the “rsem-calculate-expression” function. Differential expression analysis was performed using the Bioconductor package DESeq2 version 1.16.1 (*71*) to compute log_2_ fold changes and FDR-adjusted *P* values. Statistical significance was determined at an FDR threshold of 0.05. Data were analyzed for molecular and functional pathway enrichment using the Ingenuity Pathway Analysis (IPA) tool (QIAGEN, Redwood City, CA).

For WGS, DNA samples were isolated using the QIAamp DNA kit (QIAGEN) and sequenced using a HiSeq X (Illumina). On average, this yielded 142.72 Gb of genomic sequence per sample and an average genome-wide sequencing depth of 28.5× coverage (range, 24.65 to 32.48×). Sequencing data were processed using the GATK (Genome Analysis Toolkit) Best Practices Workflow (https://software.broadinstitute.org/gatk/best-practices/) (*72*) to produce bam files for downstream processing. The per-sample preprocessing steps included mapping reads to genome build hg38 of the human reference using Burrows-Wheeler Aligner (BWA)-maximal exact matches (MEMs) (*73*) and SAMtools (*74*), marking duplicates with Picard (https://broadinstitute.github.io/picard/) and base quality score recalibration with GATK version 3.6. Somatic mutations and Indel (i.e., insertions/deletions) were called and filtered using Mutect2 implemented in GATK version 3.6 (https://software.broadinstitute.org/gatk/) and annotated with ANNOVAR (*75*). Tumor-specific (somatic) copy number alterations were detected using VarScan2 (*76*) (http://varscan.sourceforge.net/) and the circular binary segmentation algorithm (*77*) implemented in the R Bioconductor library DNAcopy (http://bioconductor.org/packages/release/bioc/html/DNAcopy.html). Mutational processes were delineated using the R package “deconstructSigs” version 1.8.0 (*48*), and functional aneuploidy was calculated as described previously (*47*).

### Statistical methods

Primary statistical analyses of differences between means included *t* test for two-sample comparisons and analysis of variance (ANOVA) with Sheffe’s post hoc test for multiple comparisons using SPSS (IBM, Armonk, NY, USA) and GraphPad Prism software (GraphPad Software, La Jolla, CA, USA). Log-transformed tumor volumes were modeled in a linear mixed effects model with the fixed effects of genotype and day and their interaction and random effects (in slope and intercept) of tumor nested within the animal. The model was used to quantify and compare the relative tumor growth rates between NSG and NSG-Pro mice. The slopes of the linear growth curves fitted to the log-transformed tumor volumes correspond to the exponential growth rates for the raw (not log-transformed) tumor volumes. Tumor growth curves analysis was performed in R (R Foundation for Statistical Computing, Vienna, Austria).

## Notes

View/request a protocol for this paper from *Bio-protocol*.
