# Supplementary material for: NSG-Pro mouse model for uncovering resistance mechanisms and unique vulnerabilities in human luminal breast cancers
Source: Sci Adv. 2021 Sep 15;7(38):eabc8145. doi: 10.1126/sciadv.abc8145 (PMC8443188; doi:10.1126/sciadv.abc8145)
Supplement: Supplementary file 1 — Figs. S1 to S4 Legends for tables S1, S3 to S9 Tables S2 and S10 [file sciadv.abc8145_sm.pdf]

Science Advances
AAAS

Supplementary Materials for

NSG-Pro mouse model for uncovering resistance mechanisms and unique vulnerabilities in human luminal breast cancers

Yunguang Sun, Ning Yang, Fransiscus E. Utama, Sameer S. Udhane, Junling Zhang, Amy R. Peck, Alicia Yanac, Katherine Duffey, John F. Langenheim, Vindhya Udhane, Guanjun Xia, Jess F. Peterson, Julie M. Jorns, Marja T. Nevalainen, Romain Rouet, Peter Schofield, Daniel Christ, Christopher J. Ormandy, Anne L. Rosenberg, Inna Chervoneva, Shirng-Wern Tsaih, Michael J. Flister, Serge Y. Fuchs, Kay-Uwe Wagner, Hallgeir Rui*

*Corresponding author. Email: hrui@mcw.edu

Published 15 September 2021, *Sci. Adv.* **7**, eabc8145 (2021)
DOI: 10.1126/sciadv.abc8145

The PDF file includes:

Figs. S1 to S4
Legends for tables S1, S3 to S9
Tables S2 and S10

Other Supplementary Material for this manuscript includes the following:

Tables S1, S3 to S9

Figure S1

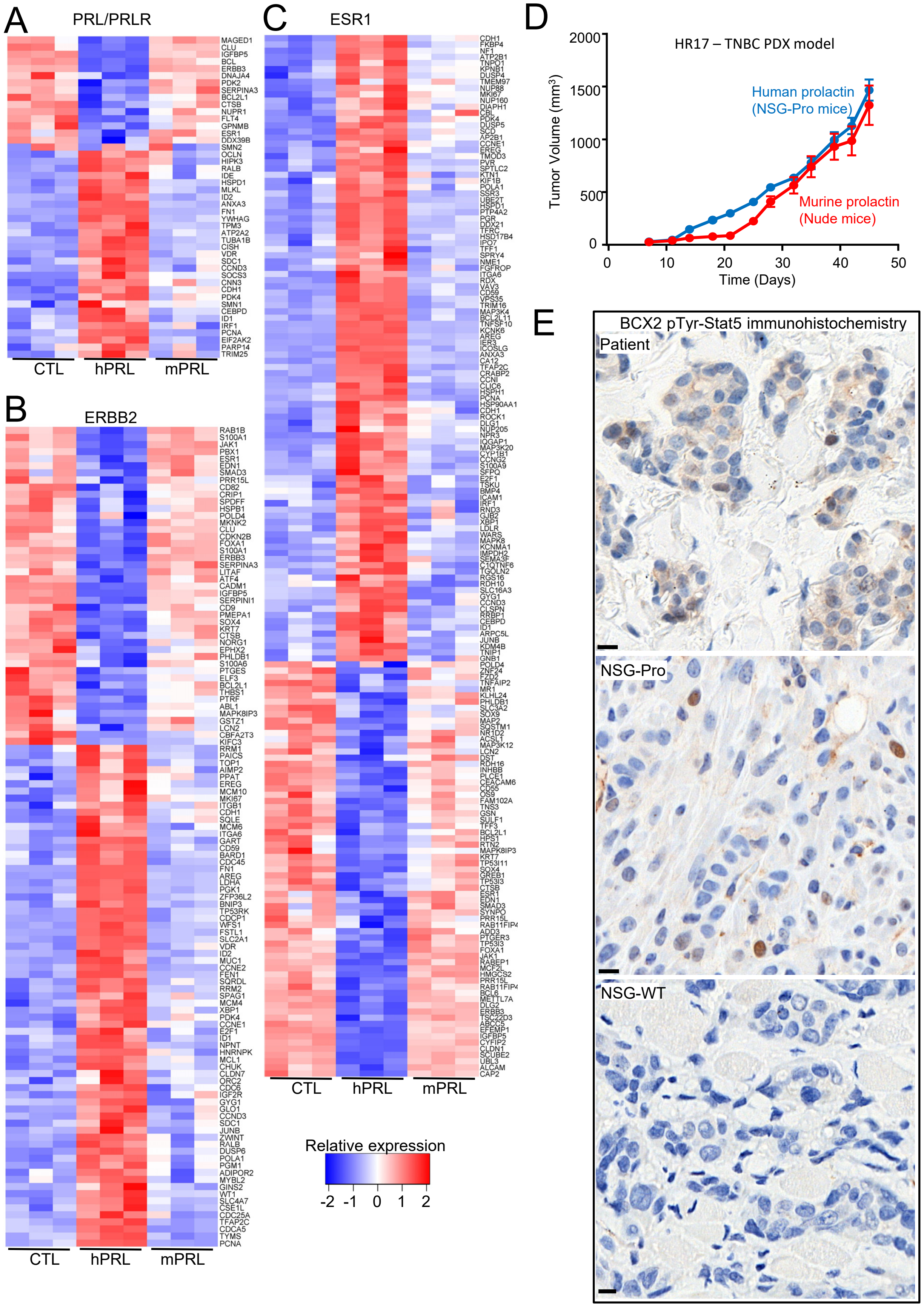
A
PRL/PRLR
CTL
hPRL
mPRL
B
ERBB2
CTL
hPRL
mPRL
C
ESR1
CTL
hPRL
mPRL
Relative expression
-2 -1 0 1 2
D
HR17 – TNBC PDX model
Tumor Volume (mm3)
2000
1500
1000
500
0
0
10
20
30
40
50
Time (Days)
Human prolactin
(NSG-Pro mice)
Murine prolactin
(Nude mice)
E
BCX2 pTyr-Stat5 immunohistochemistry
Patient
NSG-Pro
NSG-WT

Fig. S1. Human prolactin modulation of gene pathways in T47D breast cancer cells.

A) Heat map of human PRL/PRLR pathway transcripts modulated in cultured T47D cells exposed to hPRL or mPRL vs. control (CTL) for 12 h. Related to Figure 1B.

B) Heat map of human ERBB2 pathway transcripts modulated in cultured T47D cells exposed to hPRL or mPRL vs. control (CTL) for 12 h. Related to Figure 1B.

C) Heat map of human ESR1 pathway transcripts modulated in cultured T47D cells exposed to hPRL or mPRL vs. control (CTL) for 12 h. Related to Figure 1B.

D) Growth of breast cancer PDX model HR17 (PRLR-, ER-, PR-, ERBB2-) in prolactin-humanized mice (NSG-Pro) or non-humanized mice (nude). Related to Figure 1H-J.

Figure S2

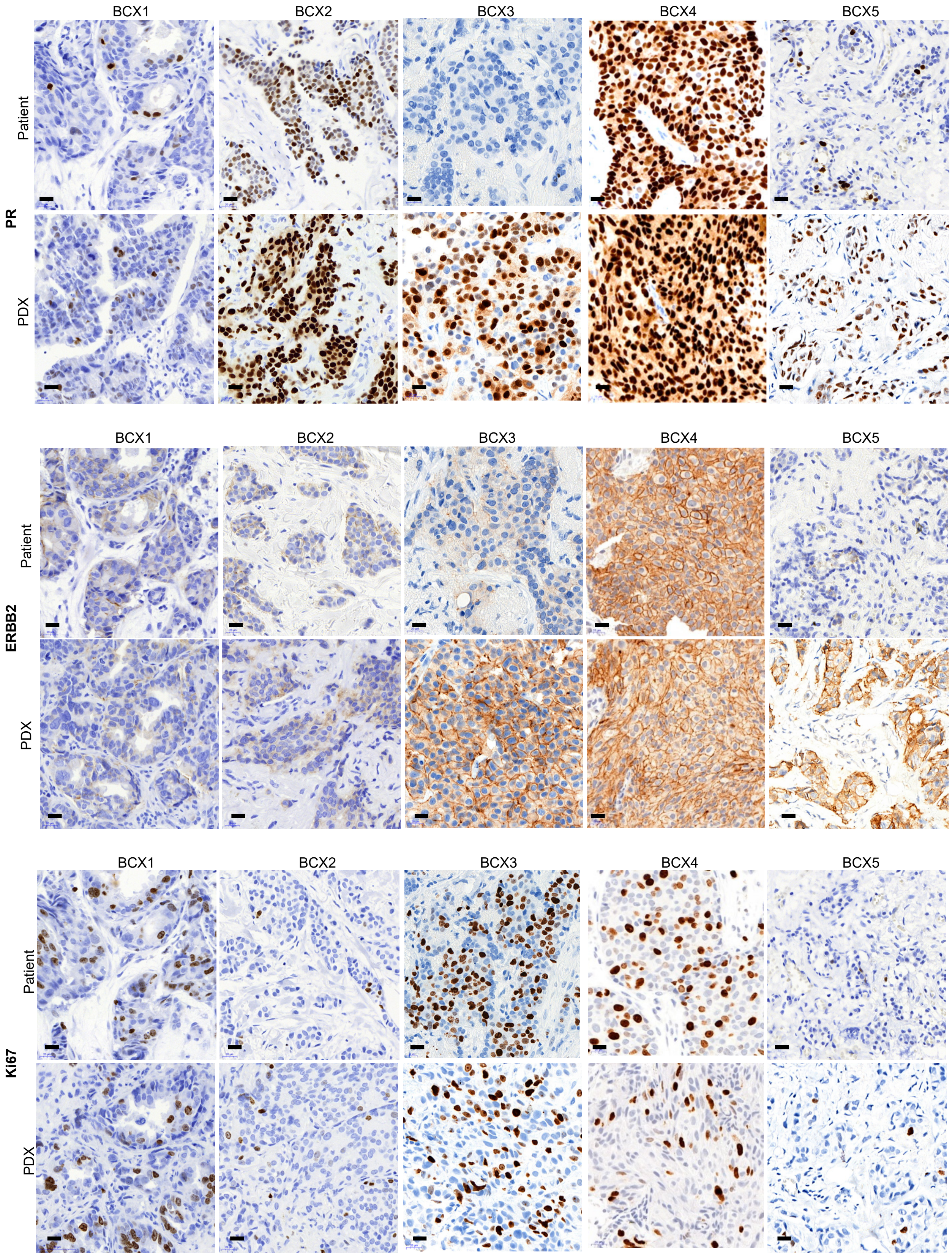

Fig. S2. Tumor markers in ER-positive patient-derived xenograft models of breast cancer

Immunohistochemistry of progesterone receptor (PR), ERBB2, or Ki67 in original patient tumors and PDX tumor models BCX1-5 grown in NSG-Pro mice. Related to Figure 2A,B.

Figure S3

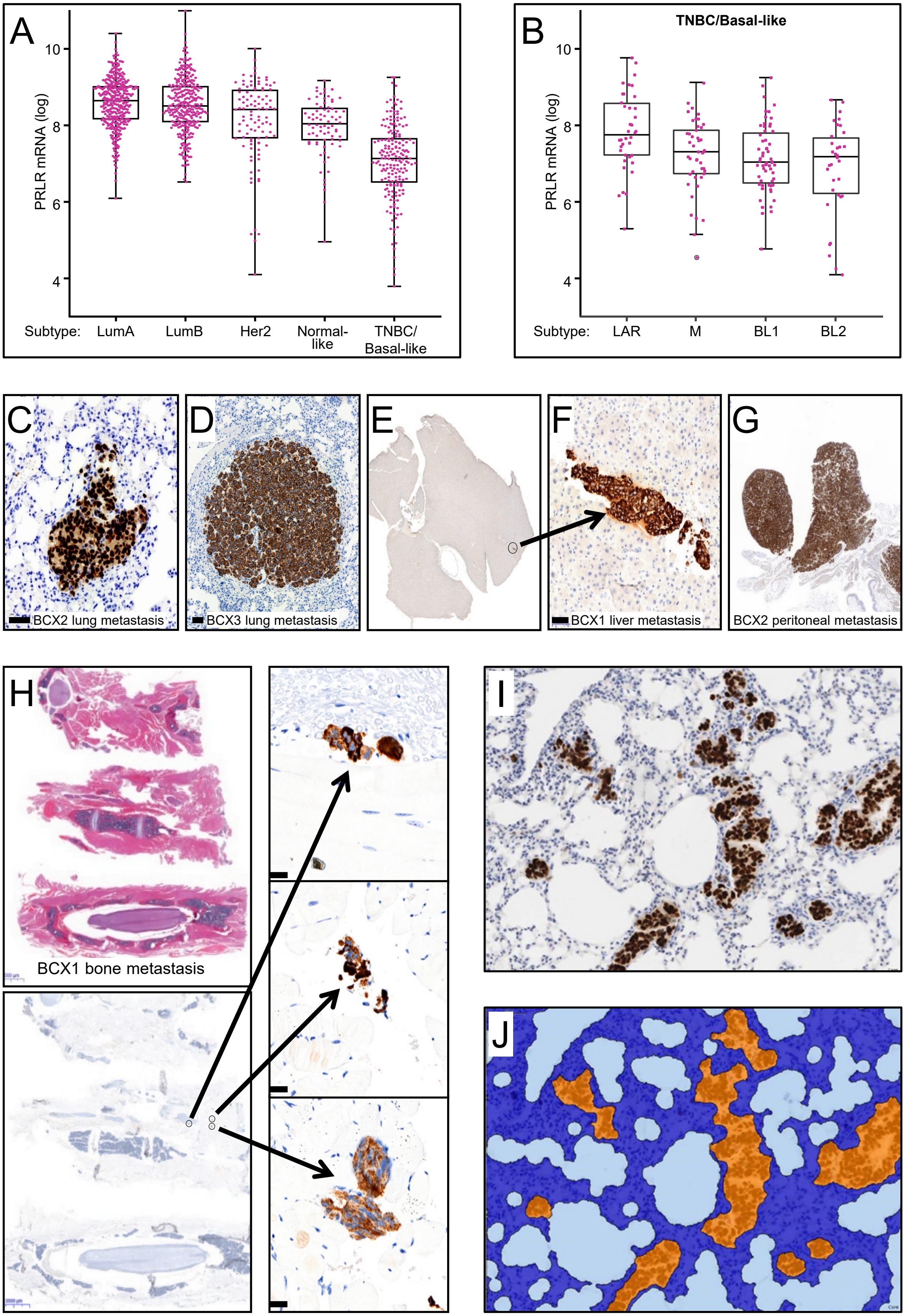
A
PRLR mRNA (log)
10
8
6
4
Subtype:
LumA
LumB
Her2
Normal-like
TNBC/ Basal-like
B
TNBC/Basal-like
PRLR mRNA (log)
10
8
6
4
Subtype:
LAR
M
BL1
BL2
C
BCX2 lung metastasis
D
BCX3 lung metastasis
E
F
BCX1 liver metastasis
G
BCX2 peritoneal metastasis
H
BCX1 bone metastasis
I
J

Fig. S3. Distant metastases of ER-positive PDX models of breast cancer. Related to Figure 3.

A) Box plot of *PRLR* transcript levels in breast cancer subtypes based on TCGA data.

B) Lung metastasis in BCX2 PDX tumor-bearing mice. Scale bar=50μm.

C) Lung metastasis in BCX3 PDX tumor-bearing mice. Scale bar=50μm.

D) Liver metastasis in BCX1 PDX tumor-bearing mice (low power).

E) Liver metastasis in BCX1 PDX tumor-bearing mice (high power). Scale bar=50μm.

F) Peritoneal metastases in BCX2 PDX tumor-bearing mice. Scale bar=500μm.

G) Bone metastases in BCX1 PDX tumor-bearing mice (H&E image and matching immunohistochemistry image with high power insets showing metastases. Scale bars=20μm.

H) Lung metastases in BCX1 PDX tumor-bearing mice as shown by anti-human specific protein marker (brown).

I) Lung metastases in BCX1 PDX tumor-bearing mice as identified by Tissue Studio quantitative image analysis software.

Figure S4

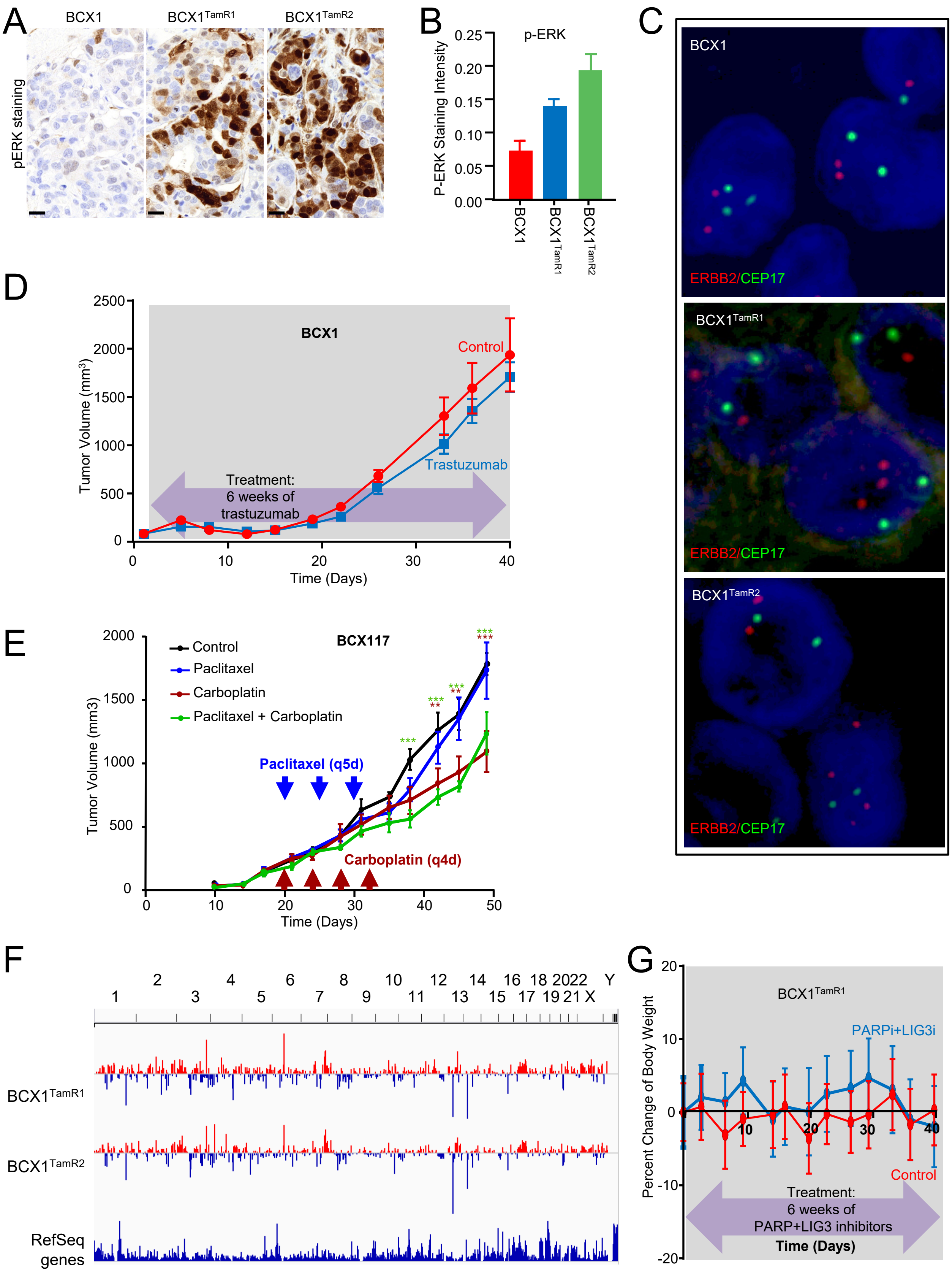

A
BCX1
BCX1TamR1
BCX1TamR2
pERK staining
B
p-ERK
P-ERK Staining Intensity
0.25
0.20
0.15
0.10
0.05
0.00
BCX1
BCX1TamR1
BCX1TamR2
C
BCX1
ERBB2/CEP17
BCX1TamR1
ERBB2/CEP17
BCX1TamR2
ERBB2/CEP17
D
BCX1
Control
Trastuzumab
Treatment:
6 weeks of
trastuzumab
Tumor Volume (mm³)
Time (Days)
E
BCX117
Control
Paclitaxel
Carboplatin
Paclitaxel + Carboplatin
Paclitaxel (q5d)
Carboplatin (q4d)
Tumor Volume (mm3)
Time (Days)
F
BCX1TamR1
BCX1TamR2
RefSeq
genes
G
BCX1TamR1
PARPi+LIG3i
Control
Percent Change of Body Weight
Treatment:
6 weeks of
PARP+LIG3 inhibitors
Time (Days)

Fig. S4. Tamoxifen-resistant BCX1 PDX tumors upregulate ERBB2 and Erk1/2 signaling and respond to trastuzumab without *ERBB2* gene amplification

A) Representative immunohistochemistry of phospho-Erk1/2 (pErk; brown) with quantification (bar graph) in BCX1^{TamR1} and BCX1^{TamR2} vs. BCX1 tumors. Scale bars=20 µm. Related to Figure 5A-G.

B) Bar graph of quantified phospho-Erk1/2 (pErk) levels in BCX1, BCX1^{TamR1} and BCX1^{TamR2} tumors. Related to Figure 5C-G.

C) Representative *ERBB2* FISH images of BCX1, BCX1^{TamR1} and BCX1^{TamR2}. Related to Figure 5H-I.

D) Tumor volumes of BCX1 PDX xenografts in NSG-Pro mice treated with trastuzumab or vehicle for 6 weeks. Related to Figure 5H-I.

E) Tumor volumes of BCX117 PDX xenografts in NSG-Pro mice treated with two different chemotherapeutic agents, documenting tumor resistance to paclitaxel but sensitivity to carboplatin, consistent with pharmacological the responsiveness pattern of the disease in the patient. Related to Figure 5H-I.

F) Visualization of up- or down-regulated human transcripts in relation to chromosomal location in tamoxifen-resistant sub-lines BCX^{TamR1} or BCX1^{TamR2} compared to estrogen-dependent BCX1 parental PDX line grown in NSG-Pro mice. Related to Figure 6A-I.

G) Body weight monitoring of mice during treatment with vehicle (Control) or combined PARP inhibitor talazoparib plus LIG3 inhibitor (PARPi/LIG3i). Related to Figure 7.

Supplementary Tables

Table S1 (Separate .xlsx file)
RNA-Seq analysis of T47D cells treated with human or mouse prolactin. Related to Figure 1B.

Table S2
Take rates of patient-derived xenografts of primary tumors in NSG-Pro mice by major subtype. Related to Figure 2A,B.

Breast Cancer	N	Tumor Take	Take Rates
ER-positive	65	28	43.1%
TNBC	7	4	57.1%
ERBB2-enriched	5	2	40.0%
Total transplanted	77	34	44.2%

Table S3 (Separate .xlsx file)
RNA-Seq analysis of BCX1 tumors grown in NSG-Pro mice compared with NSG mice. Related to Figure 2C,E,G.

Table S4 (Separate .xlsx file)
RNA-Seq analysis of BCX2 tumors grown in NSG-Pro mice compared with NSG mice. Related to Figure 2D,F,H.

Table S5 (Separate .xlsx file)
Analysis of somatic mutations in BCX1, $BCX1^{TamR1}$, $BCX1^{TamR2}$. Related to Figure 4G.

Table S6 (Separate .xlsx file)
Copy number variant analysis of the BCX1, $BCX1^{TamR1}$, $BCX1^{TamR2}$ relative to germline. Related to Figure 6A.

Table S7 (Separate .xlsx file)
Copy number variant analysis of the $BCX1^{TamR1}$, $BCX1^{TamR2}$ relative to BCX1. Related to Figure 6B.

Table S8 (Separate .xlsx file)
RNA-Seq analysis of human gene expression in $BCX1^{TamR1}$ compared to BCX1. Related to Figure 6J,K.

Table S9 (Separate .xlsx file)
RNA-Seq analysis of human gene expression in $BCX1^{TamR2}$ compared to BCX1. Related to Figure 6J,K.

Table S10

Concordance between primary patient tumor and PDX models BCX2 and BCX3 with respect to single nucleotide somatic mutations in genes highly relevant for primary breast cancer. Genomic DNA was available from patient tumors that yielded BCX2 and BCX3 PDX models and were examined for mutations in *ATM, BARD1, BRCA1, BRCA2, BRIP1, CBFB, CDH1, CHEK2, EPCAM, FANCC, FANCM, GATA3, MAP3K1, MLH1, MSH2, MSH6, MUTYH, NBN, NF1, PALB2, PI3K, PMS2, POLD1, PTEN, RAD51C, RAD51D, RECQL, SF3B1,TP53*. Exonic mutations were 100% concordant between primary tumors and the derived PDX. Mutated genes are listed for each PDX model.			
PDX	**Pathogenic*** (amino acid changes noted)	**Missense** (likely non-pathogenic)	**Synonymous**
BCX2	BRCA1(p.S509G*; p.S1566G*; p.S1613G*; p.S1634G)	BRIP1, EPCAM, MSH6, PMS2, RAD51D	CDH1,NF1
BCX3	TP53(p.P33R*; p.P72R*)	ATM, BARD1, BRCA1, BRCA2, BRIP1, EPCAM, FANCM, MLH1, NBN, PALB2, PMS2, POLD1, RECQL	CDH1, GATA3, MAP3K1,NF1, RAD51D
